# How do associations between healthy life expectancy and risk factors vary across small geographic areas in a UK integrated care system? Cross-sectional study

**DOI:** 10.1136/bmjopen-2025-108114

**Published:** 2026-07-13

**Authors:** Max Bachmann, Mike Saunders, Zillur Rahman Shabuz, Alice M Dalton, Oby Otu Enwo, Julii Brainard, Charlotte E L Jones, Amanda Burke, Mary Onoja, Aisling Ponzo, Stephanie Howard Wilsher, Sarah Gentry, Nicholas Steel

**Affiliations:** 1 Norwich Medical School, University of East Anglia, Norwich, UK; 2 Health Economics Consultancy, University of East Anglia, Norwich, UK

**Keywords:** PUBLIC HEALTH, Primary Prevention, Risk Factors, Health policy, Quality of Life

## Abstract

**Abstract:**

**Objectives:**

Local public health organisations require information about local variations in healthy life expectancy (HLE) and associated risks to inform decisions about how and where to intervene to improve HLE, a key indicator of population health. We aimed to estimate both HLE and levels of risk in small areas and quantify associations between them.

**Design:**

Cross-sectional population-based study.

**Setting:**

Norfolk and Waveney Integrated Care System.

**Population:**

128 Middle Layer Super Output Areas and eight Lower Tier Local Authority Areas.

**Outcome measures:**

HLE (estimated using self-reported health status from the 2021 UK Census) in each Middle layer Super Output Area and levels of 10 risk factors (selected based on existing evidence of association with lower life expectancy or self-reported health and availability of local risk information): index of multiple deprivation; weekly net income; urban area; diet not meeting five portions of fruit and vegetables on a usual day; physical inactivity; older person living alone; falls admissions rate; alcohol mortality rate; road casualties and air pollution.

**Main results:**

HLE in 2021 was 66.5 years for men (range 52–73) and 67.5 years for women (range 56–74). The difference between areas was 21 years for men and 18 years for women. Higher income was strongly associated with all healthy life expectancies: £100 higher weekly income was associated with 4.4 (95% confidence limits 3.5 to 5.2) and 4.6 (3.8 to 5.4) years greater HLE at birth in males and females respectively and with 1.7 (1.3 to 2.2) and 2.0 (1.5 to 2.5) greater HLE at age 65. Higher percentage of older adults living alone was associated with lower HLE at birth in males and females. Physical inactivity was associated with lower HLE at 65 in males and at birth in females.

**Conclusions:**

This approach uses standard methods and publicly available data to estimate both HLE and risk exposures in small areas to find areas with low life expectancy and high risks, where local organisations may prioritise the implementation of cost-effective interventions. It could be replicated in other areas to target interventions and inequalities. More accurate data on risk exposures in small areas would allow a broader range of risk factors, including smoking, to be considered.

**STRENGTHS AND LIMITATIONS OF THIS STUDY**

- Healthy life expectancy was estimated for Middle layer Super Output Areas using Sullivan’s method for comparability with official results for larger areas from the Office for National Statistics.
- Risk factor selection was based on robust published evidence of association with poor health outcomes.
- Data and methods are in the public domain and this method could be replicated in other regions.
- Accurate information on smoking and some other risk factors was not available for small areas.
- Estimating healthy life expectancy from Middle layer Super Output Areas for a single year, rather than a rolling average, is a trade-off between timeliness and precision, and is not recommended for populations of less than 5000.

## Introduction

 Healthy life expectancy (HLE) is a key indicator of population health in many countries.^1^ The current UK Government made a manifesto commitment to halve the gap in HLE between the richest and poorest regions in England, following the previous government’s aim to gain 5 years of HLE by 2035.^2 3^ HLE in England varies by sex, place and deprivation, with a gap of almost 20 years between the most and least deprived areas.^4^ Inequalities in HLE in England have been reported consistently over two decades, using data from the 1991 Census,^5^ the 2001 Census^6^ and the 2011 Census.^7^ To achieve improvements in HLE, local leaders need to understand the variations in drivers of HLE at small areas, and the Office for National Statistics (ONS) recommended a focus on the drivers of both self-reported health and mortality to improve understanding of HLE and inform policy.^8^

ONS produces HLE for English regions annually, based on 3-year rolling averages,^1^ and estimates HLE at birth for Middle layer Super Output Areas (MSOAs) for 5-year periods centred on the census year.^9^ The most recent release of HLE for small areas in the UK was in 2015 and covers 2009–2013, with no HLE data yet available for any release based on the most recent 2021 census.^9^ Previous analyses have found associations between shorter life expectancy (LE) and risk factors at national level, including higher alcohol intake, higher rates of smoking and obesity and physical inactivity,^810^,^12^ but there have been no recent analyses of such associations at small area level. There is therefore a need for local organisations to be able to estimate HLE for small areas more recently than 2013, and to compare local HLE with locally varying levels of exposure to health risks that are known to be linked to poor health outcomes.

Norfolk and Waveney (N&W) Integrated Care System in the East of England is a partnership between National Health Service organisations, local government and voluntary organisations which aims to help the population of a million people lead longer and healthier lives. This geographical area, with a range of deprivation and a mix of rural and urban areas, was used as an example of how local information on HLE and risk factor exposure can be estimated and compared. The objectives were to estimate HLE and the levels of risk factors for poor health for men and women at birth and age 65 years in all MSOAs in N&W in 2021, and to quantify associations between specific risk factors and HLE.

## Methods

This cross-sectional ecological study uses a standard approach to estimating healthy life expectancies in MSOAs in N&W. Associations between HLEs and risk factors for poorer health and mortality were estimated using correlation coefficients and linear regression. Data pertain to 2017–2021. N&W was divided into eight Lower Tier Local Authority (LTLA) areas and 128 MSOAs in 2021. The population of an MSOA typically ranges from 5000 to 15 000 people,^13^ a size that could provide granularity while preserving adequate precision.^14^ All data were anonymised and available in the public domain at diverse geographic resolutions and time points.

### Outcome

HLE is the number of years a person may expect to live in good health if current age-specific population morbidity and mortality rates were constant throughout their life. We estimated HLE by first estimating LE and then adjusting LE by the proportion of the population in each age and sex stratum who assessed their health as either ‘very good’ or ‘good’ in response to the national Census question on general health. Census respondents were asked to assess their general health on a 5-point scale: ‘very good’, ‘good’, ‘fair’, ‘bad’ or ‘very bad’.^15^ This is the method used by the ONS.^1^ We estimated HLE at the MSOA level and the units are years.

We estimated HLE at birth and age 65 years (the standard ages used by ONS^16^) using the Sullivan method (the standard method used by ONS^1^) with an HLE calculation template from the Office for Health Improvement and Disparities.^17^ The Sullivan method combines Census data on self-assessed general health with LE to calculate the average number of years lived in a given state of health from a given age.^1^ Census data on the population size, number of deaths and proportion of the population self-reporting good or very good health were obtained by age, sex and MSOA.^18^ We verified that the total person-years per MSOA were at least 5000 and that there were no age bands with zero deaths, as a requirement for calculating HLE.^14^ HLE cannot be estimated if there are zero deaths in the oldest age group, because the calculation results in an infinite HLE. We therefore followed the recommendation of Eayres and Williams^19^ to replace the zero-death rate in the oldest age group in an area with the average death rate of the surrounding areas. In this case, we replaced it with the average death rate for age 90 and over for all MSOAs in the study. Census self-reported health data were for the complete population and not from a sample, so the age adjustment factor and design effect adjustments were not used (by altering these values to 1). We assessed the precision of HLE estimates using 95% CIs. Our HLE estimates are suitable for comparing populations but are not projections or forecasts.

### Risk factors

HLE is affected by both mortality and self-reported health, and we selected risk factors for both categories. Risk factors for lower LE were selected from risk–outcome pairs that met the criteria for convincing or probable evidence reported in the Global Burden of Disease Study,^20^ informed by risk factors for mortality in the UK population.^21^ Risk factors for poorer self-reported health were selected from a literature review^8^ and the 2019 Health Profile for England, which included factors associated with wider determinants of health.^22^ Risk factors were excluded if information was not available for the included geographic areas. The risk factors of interest were urban versus rural location, mean weekly net household income after housing costs, percentage of adults aged over 66 living alone, percentage of adults who were physically inactive, percentage of adults not meeting the five a day dietary target, male and female alcohol mortality rates (as indicators of unhealthy alcohol consumption), falls admission rates, air pollution (particulate concentration in μg/m^3^) and annual number of road casualties. These risk factors were assumed to be proxy indicators of factors that could directly affect the health of individuals living within each MSOA. We also considered household income and percentage of older adults living alone to be complex indicators of social position that could affect multiple other health-related factors, acting at individual, household and societal levels. Data were available at MSOA level except for falls, physical inactivity, diet and alcohol admissions, for which information originated at LTLA level, which is larger than MSOAs. LTLA data were applied equally to all MSOAs within the LTLA boundary. MSOAs are built from groups of contiguous Lower layer Super Output Areas (LSOA).^13^ LSOA-derived data were accurately aggregated from LSOA to their respective MSOA. Risk factors were excluded if accurate data were not available. We examined scatter graphs for heteroscedasticity and examined histograms of HLEs for normality. Risk factors and data sources are described in online supplemental material and online supplemental table S1.

### Statistical methods

Statistical analyses were conducted at MSOA level. We estimated the mean, SD and range of each HLE outcome across all MSOAs. We aimed to estimate associations between HLEs and risk factors for poorer health and mortality. We first calculated pairwise Pearson correlation coefficients between each HLE estimate and each risk factor. We then constructed four linear regression models, with HLE (at birth and at age 65 in males and females) as outcomes and with all risk factors as covariates. To assess collinearity of risk factors, we estimated variance inflation factors (VIFs) after each regression model. As a sensitivity analysis, because household income was most strongly associated with HLEs and was also strongly associated with several risk factors, we repeated the four regression models, omitting income as a covariate. To assess the appropriateness of using linear regression models, we examined histograms of the residuals of each model. A p value of <0.05 was used to indicate a statistically significant association. Statistical analyses were conducted with Stata V.19 statistical software.^23^ We did not do prior sample size or power calculations because the sample size was pre-determined and there was no previous evidence to inform assumptions about the magnitude of parameters required for the calculations. We produced maps to visualise HLE estimates and risk factors in 2021, using ArcGIS Pro V.3.0.1.^24^

## Results

The unweighted average HLE at birth in 2021 was 66.5 years for men (range 52–73) and 67.5 years for women (range 56–74) (table 1).

**Table 1:** Healthy life expectancy and life expectancy in Norfolk and Waveney Middle layer Super Output Areas (2021) at birth and age 65

	At birth (years)		Age 65 (years)	
	Male	Female	Male	Female
	**Healthy life expectancy**			
Mean (SD)	66.5 (4.3)	67.5 (3.9)	10.9 (1.8)	12.2 (1.9)
Range	52.0–73.0	56.0–74.0	6.0–16.0	5.0–15.0
	**Life expectancy**			
Mean (SD)	82.0 (3.3)	85.2 (3.1)	18.9 (2.1)	21.3 (2.3)
Range	71.0–89.0	75.0–92.0	14.0–27.0	13.0–27.0

### Small area variation in HLE and risk factor exposure

HLE at age 65 and risk factor information were tabulated for each MSOA by deciles of risk factor levels, for women (table 2), men (online supplemental table S2) and men and women combined (online supplemental table S3). To aid identification of clusters of risk factors and low HLE, these tables are coloured by decile with red for short HLE and high risk and blue for long HLE and lower risk. A risk factor decile of 1 indicated an MSOA in the 10% with the greatest level of the specific risk factor and 10 indicated an MSOA in the 10% with the lowest level. If data originated from one of the eight Lower Tier Local Authorities (LTLA), risks were ranked by LTLA level, with one meaning an MSOA is within the LTLA with the highest level and eight the lowest.

**Table 2:** Healthy life expectancy (HLE) at age 65 for women in 2021 and risk factors for the Middle Layer Super Output Areas (MSOAs) with the 15 lowest and highest HLE estimates

Lower tier local authority	MSOA name	HLE at 65 years, women (years)	Falls admissions, women (rate)	Not eating 5-a-day (%)	Older person living alone (%)	Physical inactivity (%)	Road casualties (n)	Income after housing (weekly, GBP)
King’s Lynn and West Norfolk	North Lynn	5	2286	43	54	31	7	425
Great Yarmouth	Yarmouth Parade	7	1245	50	61	29	9	390
East Suffolk	Gunton West	8	1425	35	53	20	0	448
Great Yarmouth	Gorleston West	8	1245	50	50	29	6	469
Great Yarmouth	Yarmouth North	8	1245	50	51	29	5	456
Breckland	Thetford South	9	2175	43	51	22	10	496
East Suffolk	Lowestoft Central	9	1425	35	57	20	0	462
East Suffolk	Lowestoft Harbour and Kirkley	9	1425	35	66	20	0	458
Great Yarmouth	Gorleston North	9	1245	50	54	29	5	454
Great Yarmouth	Yarmouth Central and Northgate	9	1245	50	57	29	4	433
Breckland	Mundford, Weeting and Forest	10	2175	43	37	22	2	567
East Suffolk	Normanston and Oulton Broad East	10	1425	35	48	20	0	508
East Suffolk	Pakefield North	10	1425	35	52	20	0	475
Great Yarmouth	Southtown and Cobholm	10	1245	50	54	29	9	477
King’s Lynn and West Norfolk	Gaywood Chase and Old Gaywood	10	2286	43	51	31	1	475
South Norfolk	Wymondham East and Spooner Row	14	1833	39	45	19	2	608
South Norfolk	Wymondham West	14	1833	39	54	19	2	548
Breckland	East Harling, Garboldisham and Kenninghall	15	2175	43	35	22	6	619
Broadland	Brundall and Cantley	15	1531	39	40	22	7	637
Broadland	Spixworth and St Faiths	15	1531	39	42	22	4	554
East Suffolk	Southwold, Reydon and Wrentham	15	1425	35	46	20	0	602
Great Yarmouth	Gorleston South and Beach	15	1245	50	46	29	7	560
King’s Lynn and West Norfolk	Brancaster, Burnham Market and Docking	15	2286	43	42	31	5	619
King’s Lynn and West Norfolk	Wootton	15	2286	43	36	31	4	673
North Norfolk	Overstrand, Roughton and the Runtons	15	1779	39	38	16	4	581
North Norfolk	Sheringham	15	1779	39	45	16	5	621
North Norfolk	Wells and Blakeney	15	1779	39	46	16	2	613
Norwich	Eaton	15	2093	48	47	19	2	669
South Norfolk	Thurlton, Haddiscoe and Geldeston	15	1833	39	35	19	5	587
South Norfolk	Trowse, Poringland and Stoke Holy Cross	15	1833	39	42	19	4	654

Ordered from lowest to highest HLE at age 65 for women. HLE estimates in the worst decile are highlighted in red, and those in the best decile in blue. Risk factor estimates in the worst decile are highlighted in red and those in the best decile in blue.

The general pattern shown in the tables is that areas with the shortest HLE also experienced the highest exposure to risk factors and vice versa. However, the variation between MSOAs reveals a more complex picture with some areas with shorter HLE experiencing lower exposure to one or two risk factors, and some areas with longer HLE experiencing high exposure to one or two risk factors, in contrast to the general pattern.

**Figure 1:**
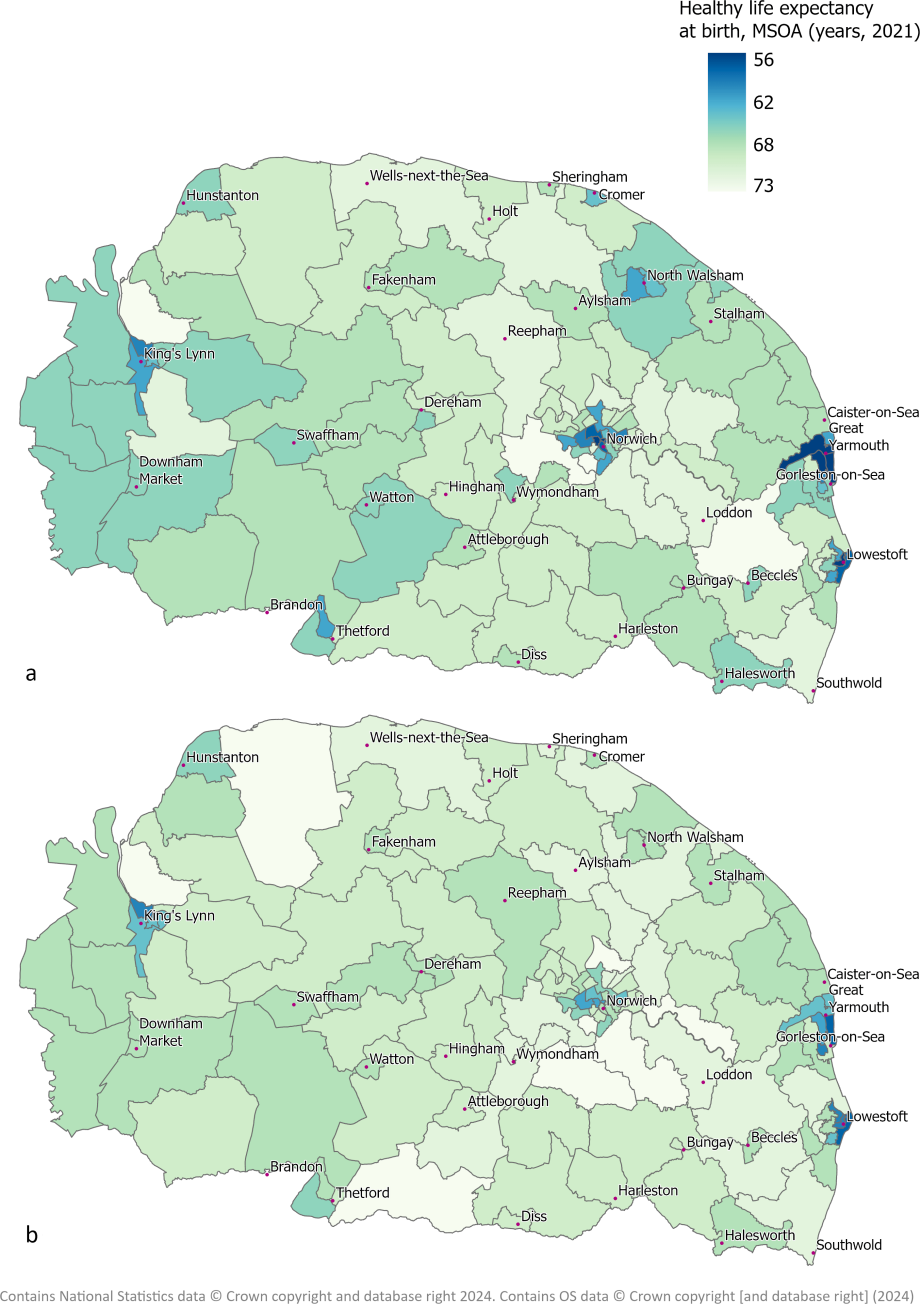
Estimated healthy life expectancy at birth for men (**a**) and women (**b**) in Norfolk and Waveney by MSOA in 2021. MSOA, Middle layer Super Output Areas.

**Figure 2:**
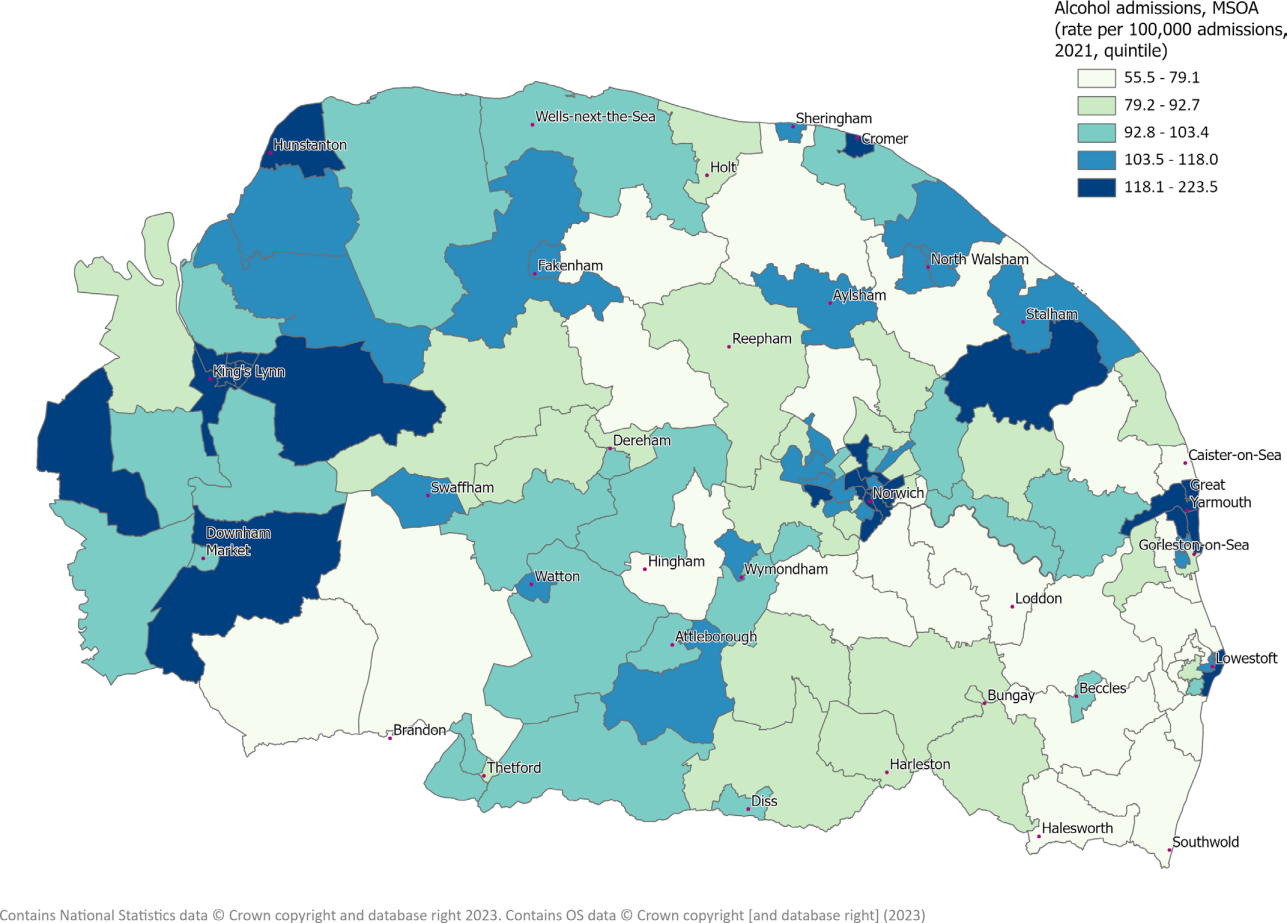
Alcohol admissions rate in Norfolk and Waveney by MSOA in 2021. MSOA, Middle layer Super Output Areas.

HLE at birth in 2021 was lower in MSOAs near Great Yarmouth and Lowestoft on the coast, and in urban areas (Norwich and King’s Lynn) (figure 1). Maps show how areas of shorter HLE (figure 1 and onlinesupplemental figures S1  S2) also usually experienced higher levels of associated risk factors (figure 2 and onlinesupplemental figures S3S6).

### HLE and associated risk factors

Pairwise correlation between HLEs and risk factors are shown in online supplemental table S4. HLEs were most strongly associated with weekly net income after housing (Pearson’s R 0.76–0.86). ‘Older adults living alone’ was strongly associated with several other risk factors. Poor diet was strongly associated with alcohol mortality, and air pollution was strongly associated with urban location. Scatter graphs and histograms of HLEs showed approximate heteroscedasticity and normality respectively.

In linear regression models (table 3 (females) and table 4 (males)), higher income was strongly associated with all HLEs: £100 higher weekly income was associated with 4.4 (95% confidence limits 3.5 to 5.2) and 4.6 (3.8 to 5.4) years greater HLE at birth in males and females, respectively, and with 1.7 (1.3 to 2.2) and 2.0 (1.5 to 2.5) years greater HLE at age 65. Higher percentage of older adults living alone was associated with lower HLE at birth in males and females. Physical inactivity was associated with lower HLE at 65 in males and at birth in females. VIFs indicated moderate collinearity of risk factors (mean VIFs 2.4–3.7) (online supplemental table S5). VIFs were highest for poor diet and alcohol mortality in males (VIFs 8.6 and 8.9), in keeping with the strong correlation between these two variables (R=0.79), and for all other risk factors VIFs were less than 5. These VIFs indicate that collinearity between covariates may have contributed to some associations being non-significant. Histograms of the models’ residuals confirmed that they were approximately normally distributed and that linear regression models were therefore appropriate.

**Table 3:** Independent associations between risk factors and years of healthy life expectancy in Norfolk and Waveney in 2021, females: multiple linear regression models with all covariates, and with income omitted

Outcome	Females at birth				Females at age 65			
Risk factor	Beta	(95% CI)		P value	Beta	(95% CI)		P value
Full models with all covariates								
Urban vs rural	0.42	−0.57	1.40	0.41	−0.03	0.31	−0.08	0.94
Adults physically inactive (%)	−0.14	−0.23	−0.04	0.01	−0.05	0.03	−1.66	0.10
Not meeting 5 a day diet (%)	0.09	−0.06	0.24	0.23	−0.02	0.05	−0.52	0.60
Age >66 living alone (%)	−0.12	−0.19	−0.05	0.00	−0.01	0.02	−0.38	0.70
Alcohol mortality	−0.08	−0.22	0.07	0.30	0.00	−0.05	0.09	0.93
Falls admission rate/100	0.05	−0.03	0.13	0.22	0.05	−0.03	0.13	0.22
Air pollution (μg/m^3^)	−0.58	−1.53	0.37	0.23	−0.41	0.30	−1.37	0.17
Road casualties (n)	−0.05	−0.16	0.07	0.41	−0.02	0.04	−0.53	0.60
Weekly net income after housing/£100	4.57	3.80	5.35	<0.001	2.03	1.48	2.48	<0.001
R square for model	0.79				0.62			
Sensitivity analysis omitting income								
Urban vs rural	−0.02	−1.45	1.41	0.98	−0.22	−0.99	0.55	0.57
Adults physically inactive (%)	−0.35	−0.48	−0.21	<0.001	−0.14	−0.21	−0.07	<0.001
Not meeting 5 a day diet (%)	0.26	0.05	0.46	0.02	0.05	−0.06	0.16	0.38
Age >66 living alone (%)	−0.32	−0.41	−0.23	<0.001	−0.10	−0.14	−0.05	0.00
Alcohol mortality	−0.31	−0.51	−0.10	<0.001	−0.10	−0.21	0.01	0.08
Falls admission rate/100	0.27	0.10	0.44	<0.001	0.11	0.02	0.20	0.02
Air pollution (μg/m^3^)	0.22	−1.15	1.59	0.75	−0.06	−0.80	0.68	0.88
Road casualties (n)	−0.19	−0.36	−0.03	0.02	−0.08	−0.17	0.00	0.06
R^2^	0.56				0.41			

**Table 4:** Independent associations between risk factors and years of healthy life expectancy in Norfolk and Waveney in 2021, males: multiple linear regression models with all covariates, and with income omitted

Outcome	Males at birth				Males at age 65			
Risk factor	Beta	(95% CI)		P value	Beta	(95% CI)		P value
Full models with all covariates								
Urban vs rural	0.65	−0.47	1.77	0.25	−0.08	−0.63	0.48	0.79
Adults physically inactive (%)	−0.04	−0.15	0.07	0.52	−0.07	−0.13	−0.02	0.01
Not meeting 5 a day diet (%)	−0.04	−0.29	0.21	0.77	0.01	−0.11	0.14	0.83
Age >66 living alone (%)	−0.19	−0.27	−0.11	<0.001	−0.03	−0.07	0.01	0.15
Alcohol mortality	0.02	−0.17	0.21	0.84	−0.02	−0.12	0.08	0.69
Falls admission rate/100	0.14	−0.02	0.30	0.08	0.05	−0.03	0.13	0.22
Air pollution (μg/m^3^)	−0.58	−1.79	0.62	0.34	−0.41	−1.01	0.19	0.18
Road casualties (n)	−0.09	−0.22	0.04	0.18	−0.05	−0.12	0.02	0.14
Weekly net income after housing/£100	4.35	3.48	5.22	<0.001	1.74	1.30	2.17	<0.001
R^2^ for model	0.77				0.67			
Sensitivity analysis omitting income								
Urban vs rural	0.23	−1.27	1.73	0.77	−0.24	−0.93	0.44	0.48
Adults physically inactive (%)	−0.22	−0.36	−0.08	<0.001	−0.15	−0.21	−0.08	<0.001
Not meeting 5 a day diet (%)	0.09	−0.25	0.43	0.61	0.06	−0.09	0.22	0.41
Age >66 living alone (%)	−0.39	−0.48	−0.30	<0.001	−0.11	−0.15	−0.07	<0.001
Alcohol mortality	−0.06	−0.32	0.20	0.66	−0.05	−0.17	0.07	0.40
Falls admission rate/100	0.27	0.06	0.48	0.01	0.10	0.002	0.20	0.05
Air pollution (μg/m^3^)	−0.22	−1.83	1.40	0.79	−0.26	−1.00	0.48	0.49
Road casualties (n)	−0.24	−0.41	−0.07	0.01	−0.11	−0.19	−0.03	0.01
R^2^	0.58				0.50			

Sensitivity analyses which omitted income from the models suggested that physical inactivity, older adults living alone, alcohol mortality, road casualties and falls admission rates were all associated with lower HLEs (table 3 (females) and table 4 (males)). However, these results are likely to be confounded by income, because income was associated with HLEs and with most risk factors (online supplemental table S4). Positive associations between fall admission rates and HLEs probably could be due to confounding by age. R^2^ values for these alternative models were substantially lower (0.41 to 0.58) than for equivalent models that included income (0.62 to 0.79) (tables3  4). Some risk factors showed no statistically significant association with HLE, notably urban versus rural location, percentage of adults not meeting the five-a-day dietary target and air pollution.

## Discussion

Males in N&W in 2021 could expect 66.5 years of healthy life, and females 67.5, compared with 61.5 and 61.9 years, respectively, in England.^16^ The difference in HLE at age 65 between the highest- and lowest-ranked areas in N&W in 2021 was 15 years for males and 10 years for females. Longer HLE was strongly associated with higher income in small areas, as well as a lower percentage of older adults living alone and lower rates of physical inactivity. The maps show areas with both low HLE and high levels of significantly associated risk factors, particularly in coastal areas such as Great Yarmouth and Lowestoft.

### Limitations

We used Sullivan’s method of HLE estimation which is widely accepted^11 25 26^ but assumes that current age-specific population morbidity and mortality will be constant throughout life, which may not be the case. We used a single-year estimate of HLE for MSOAs, whereas ONS uses a 5-year period centred around the census year to smooth the estimates because of relatively small numbers.^1^ There is a tradeoff between the timeliness of HLE estimates and improved precision, and we prioritised timeliness, given that the most recent ONS estimates are from 2013 and local public health organisations need more timely data. MSOA population sizes are at least 5000, which is the minimum size recommended for calculating LE,^14^ and so we believe that the loss in precision from estimating single-year HLE is likely to be small and represents an acceptable tradeoff for timely information until the ONS releases more precise estimates from pooled years.

The estimated coefficients for risk factors other than income have wide CIs because of the relatively small number of MSOAs. The risk factors were selected because good quality evidence links them to health outcomes, and our results show varying associations between risk factors and outcomes in different small areas but cannot show causation. In some instances, such as falls, income and poor health, reverse causality cannot be excluded. Our data are cross-sectional and although risk factors can have short-term consequences, many have influences on health over decades that might not be fully captured with these data. All estimates refer to MSOA populations and cannot be assumed to apply to all individuals within those population groups. Spatial dependencies were not tested.

If variables were not measured accurately their estimated associations would tend to be biased towards showing no association. For this reason, we excluded some publicly available data, with smoking data being a notable example. Smoking data^27^ was excluded because the survey sample size was small (range 100–380 people), the overall response rate in 2021 was low at 23% (for the Labour Force Survey, which collects the variable about smoking for the Annual Population Survey), some populations were excluded from the survey (members of armed forces if not living in private accommodation, and people living in communal housing other than NHS housing and students in halls of residence), the prevalence changed during a switch to telephone sampling and approximately 30% of responses were made by people other than the individual surveyed. Exclusion of smoking data may have led to some unmeasured confounding. More accurate data on risk exposures in small areas is needed, particularly for smoking and also for other behavioural exposures such as diet and physical inactivity.

We were unable to obtain data on certain risk factors for the area or year required, for example, infectious disease or prevention rates, and a direct measure of alcohol consumption. Most risk factors were not disaggregated by sex, and where not available, we assumed an equal distribution by sex. Comparison of risk factors was limited by differences in units of measurement, years, populations included and original area level of the data source.

### Implications for policy and practice

A long-term strategic priority to improve HLE and narrow the differences between local areas requires commitment to reducing harmful risk factors and this study has demonstrated the potential benefits of looking at risk factor variation at MSOA level. This approach could help local areas improve HLE and potentially narrow gaps between small areas, by identifying areas to target for intervention and evaluation of any impact, for example, as part of Population Health Management programmes. It may also be useful in primary care in terms of understanding risk factor priorities for practices in different areas.

The local variations in risk factor levels identified were sometimes unexpected and this approach would be useful in prompting public health teams and organisations to explore the areas with more complex risk factor pictures further. For example, the associations between HLE and physical inactivity are striking and potentially amenable to local action. Cost-effective interventions exist for risk factors identified in this study, which are suitable for implementation in small geographic areas. Marteau and colleagues suggest interventions based around four leading behavioural causes of years of life lost: tobacco use, unhealthy diet, alcohol consumption and physical inactivity.^28^ The UK Department of Health and Social Care recommended place-based and whole systems approaches to improving health and reducing inequalities, focused on disadvantaged populations, for example, for increasing physical activity and reducing obesity.^29 30^

Examples of evidence-based interventions to reduce the main risk factors in this paper include the Retirement in Action community programme. This was cost-effective in enhancing the quality of life of older adults prone to mobility issues, cost £622 per participant and saved £100 in health and social care utilisation costs in the intervention compared with the control group over 24 months.^31^ National alcohol pricing policies were found to be cost-effective in decreasing alcohol consumption and associated mortality, disease prevalence and admissions among English adults.^32^ Cost-effective local interventions include stepped-care for hazardous alcohol users aged 55 and over, which cost £171 less per quality-adjusted life year gained over 12 months relative to minimal intervention.^33^ Targeting younger populations has also been found to be cost-effective in preventing heavy drinking in the Steps Towards Alcohol Misuse Prevention Programme, which cost £426 per school and £8 per pupil aged 11–12 years.^34^

## Conclusions

We estimated male and female HLE at birth and age 65 years at MSOA level in N&W for 2021 and tested the size and direction of associations with evidence-based risk factors. This approach can inform interventions to improve HLE and narrow the gap between areas. It could be used by local authorities in other areas to monitor trends in HLE and exposure to risks, and to evaluate the impact of interventions and policies which aim to reduce the exposure of populations to harmful risks at local level, improve HLE and reduce inequality.

## Supplementary material

10.1136/bmjopen-2025-108114 online supplemental file 1

10.1136/bmjopen-2025-108114 online supplemental figure 1

10.1136/bmjopen-2025-108114 online supplemental figure 2

10.1136/bmjopen-2025-108114 online supplemental figure 3

10.1136/bmjopen-2025-108114 online supplemental figure 4

10.1136/bmjopen-2025-108114 online supplemental figure 5

10.1136/bmjopen-2025-108114 online supplemental figure 6

## Data Availability

Data are available in a public, open access repository.

## References

[1] Office for National Statistics (2025). Health state life expectancies, UK QMI: 2021 to 2023.

[2] Department for Levelling Up Housing and Communities (2022). Levelling Up the United Kingdom.

[3] The Labour Party (2024). Labour Party Manifesto 2024.

[4] Office for National Statistics (2022). Health state life expectancies by national deprivation deciles, England: 2018 to 2020.

[5] Bajekal M (2005). Healthy life expectancy by area deprivation: magnitude and trends in England, 1994-1999. Health Stat Q.

[6] Smith MP, Olatunde O, White C (2010). Monitoring inequalities in health expectancies in England - small area analyses from the Census 2001 and General Household Survey 2001-05. Health Stat Q.

[7] Marmot MAJ, Boyce T, Goldblatt P (2020). Health equity in England: the marmot review 10 years on.

[8] Office for Health Improvement & Disparities (2023). Understanding the drivers of healthy life expectancy: report.

[9] Office for National Statistics (2015). Health Expectancies at Birth for Middle Layer Super Output Areas (MSOAs), England: 2009 to 2013.

[10] Raleigh V (2022). What is happening to life expectancy in England?.

[11] Zaninotto P, Head J, Steptoe A (2020). Behavioural risk factors and healthy life expectancy: evidence from two longitudinal studies of ageing in England and the US. Sci Rep.

[12] Chudasama YV, Khunti K, Gillies CL (2020). Healthy lifestyle and life expectancy in people with multimorbidity in the UK Biobank: A longitudinal cohort study. PLoS Med.

[13] Office for National Statistics (2023). Census 2021 geographies. https://www.ons.gov.uk/methodology/geography/ukgeographies/censusgeographies/census2021geographies.

[14] Toson B, Baker A (2003). Life expectancy at birth: methodological options for small populations.

[15] Office for National Statistics (2023). General health, England and Wales: Census 2021.

[16] Office for National Statistics (2024). Healthy life expectancy in England and Wales: between 2011 to 2013 and 2021 to 2023.

[17] Office for National Statistics (2019). Health state life expectancy estimates template.

[18] Office for National Statistics (2021). Census for England and Wales.

[19] Eayres D, Williams ES (2004). Evaluation of methodologies for small area life expectancy estimation. J Epidemiol Community Health.

[20] GBD 2019 Risk Factor Collaborators (2019). Global burden of 87 risk factors in 204 countries and territories, 1990–2019: a systematic analysis for the Global Burden of Disease Study 2019. The Lancet.

[21] Steel N, Ford JA, Newton JN (2018). Changes in health in the countries of the UK and 150 English Local Authority areas 1990–2016: a systematic analysis for the Global Burden of Disease Study 2016. The Lancet.

[22] Public Health England (2019). Health profile for England: 2019.

[23] StataCorp

[24] Esri inc (2022). ArcGIS pro 3.0.1.

[25] Office for National Statistics (2017). An overview of lifestyles and wider characteristics linked to Healthy Life Expectancy in England.

[26] Lynch M, Bucknall M, Jagger C (2022). Projections of healthy working life expectancy in England to the year 2035. *Nat Aging*.

[27] Office for Health Improvement & Disparities (2023). Smoking Prevalence in adults (18+) - current smokers (APS).

[28] Marteau TM, Rutter H, Marmot M (2021). Changing behaviour: an essential component of tackling health inequalities. BMJ.

[29] Public Health England (2021). Place-based approaches for reducing health inequalities: main report.

[30] Public Health England (2020). Whole systems approach to obesity.

[31] Snowsill TM, Stathi A, Green C (2022). Cost-effectiveness of a physical activity and behaviour maintenance programme on functional mobility decline in older adults: an economic evaluation of the REACT (Retirement in Action) trial. Lancet Public Health.

[32] Purshouse RC, Meier PS, Brennan A (2010). Estimated effect of alcohol pricing policies on health and health economic outcomes in England: an epidemiological model. Lancet.

[33] Coulton S, Bland M, Crosby H (2017). Effectiveness and Cost-effectiveness of Opportunistic Screening and Stepped-care Interventions for Older Alcohol Users in Primary Care. Alcohol Alcohol.

[34] Agus A, McKay M, Cole J (2019). Cost-effectiveness of a combined classroom curriculum and parental intervention: economic evaluation of data from the Steps Towards Alcohol Misuse Prevention Programme cluster randomised controlled trial. BMJ Open.

