# Supplementary material for: How do associations between healthy life expectancy and risk factors vary across small geographic areas in a UK integrated care system? Cross-sectional study
Source: BMJ Open. 2026 Jul 13;16(7):e108114. doi: 10.1136/bmjopen-2025-108114 (PMC13365761; doi:10.1136/bmjopen-2025-108114)
Supplement: online supplemental file 1 [file bmjopen-16-7-s001.docx]

**Supplementary material**

**Risk factor information**

*Pollution - particulate matter PM 2.5mm diameter*

Background pollution for particulate matter (PM 2.5mm diameter), a pollutant which causes a range of health complications in humans(1), was modelled by Defra using a 1 km x 1 km grid resolution across the UK(2). Data on annual mean concentration of PM 2.5mm for 2021 were downloaded, imported into ArcGIS Pro version 3.0.1(3), and displayed spatially according to the coordinates in the data, to the British National Grid coordinate system. The resulting data were a grid of data points spaced 1km apart, each with a value for modelled PM 2.5mm concentration at that spatial location. The point data were spatially joined to the MSOA boundaries in Norfolk and Waveney, using the ‘Add Spatial Join’ tool. The tool was used to select all data points within 100m of each MSOA (to overcome potential edge/boundary effects(4)) and to calculate the average value of all of those points for each MSOA.

*Income*

The closest available years for income data at MSOA level were 2019-20. Data were obtained from the Office for National Statistics (ONS) and restricted to Norfolk and Waveney MSOAs. The 2019-20 file presented income per year, and was divided by 52, to provide weekly net income after housing.

*Appraisal of data quality*

If data were not available for 2021, we used the most recent year. Data were obtained stratified by sex where possible, and if not available, equal distribution by sex was assumed. The final set of risk factors was selected based on validity, reliability, availability, relevance, and time proximity to the desired year for MSOAs. Validity was assessed by reviewing whether the data item was a direct or proxy measure, and whether the sampled population included or could be considered similar to the study population. Reliability was assessed by reviewing whether data was collected, measured, and used in a consistent and transparent manner over time and place or population, and whether there were any data quality issues reported. Relevance was assessed by considering the similarity between the available data item and the evidence-base used to scope our data search.

**Supplementary tables**

- Table S1 Risk factors for low healthy life expectancy and life expectancy, data definitions and sources
- Table S2 Norfolk and Waveney MSOAs with 15 lowest and highest HLE at age 65 estimates for men in 2021, and predictor prevalence by MSOA
- Table S3 Norfolk and Waveney MSOAs with HLE estimates at birth and age 65 for men and women in 2021, and predictor prevalence by MSOA
- Table S4 Pairwise Pearson correlation coefficients for outcomes and covariates included in linear regression models
- Table S5 Variance inflation factors (VIF) for covariates included in linear regression models (Table 3)

*Table S1 Risk factors for low healthy life expectancy and life expectancy,* *data definitions and sources*

| **Category** | **Year** | **Data Item** | **Data Description** | **Area** | **Source** |
| --- | --- | --- | --- | --- | --- |
| **HLE and LE 2021** | | | | | |
| HLE and LE Calculation | 2021 | Population count | Number of people in the resident population, by age and sex | MSOA | Census 2021(5) |
|  |  | Deaths count | Number of recorded deaths, by age and sex |  |  |
|  |  | Self-reported health rating counts | Number of people by age and sex self-reporting health as good or very good on a 5-point scale ranging from very bad to very good |  |  |
| Risk factors for poor health | 2021 - 2022 | Falls admission rate | Directly standardised rate (per 100,000 population European Standard Population) of emergency hospital admissions due to falls in people aged 65 and over | LTLA | OHID Fingertips (6) |
|  | 2021 | Road traffic accident casualties (killed or injured) | Number of people killed or injured on the roads reported to police | LSOA | Department for Transport (7) |
|  | 2021 - 2022 | Physical inactivity in adults | Percentage of physically inactive adults aged 19+ | LTLA | Active Lives Survey (6) |
|  | 2019 - 2020 | Diet not meeting 5 a day on usual day | Proportion of population aged 16+ reporting they had not eaten the recommended 5 portions of fruit and vegetables a day on a usual day. Calculated using difference between total population and population reporting they eat the recommended ‘5 a day’ on a usual day. | LTLA | Active Lives Survey (6) |
|  | 2017 - 2019 | Admission episodes for alcohol-specific conditions | Admissions to hospital where either the primary diagnosis (main reason for admission) or one of the secondary (contributory) diagnoses is an alcohol-related condition (broad’ definition). Directly age standardised rate per 100,000 population (standardised to the European standard population). | LTLA | OHID Fingertips(6) |
|  | 2021 | Air Pollution | Modelled average annual concentration PM2.5 μg / m^3^ | km^2^ | DEFRA(8) |
|  | 2019-2020 | Weekly average income | Weekly net income after housing per MSOA in GBP | MSOA | ONS(9) |
|  | 2021 | Proportion of people aged 66 and over living alone | Age of Household Reference Person by Household lifestage | MSOA | Census 2021(5) |
|  | 2019 | Index of Multiple Deprivation Score | Aggregate average Index of Multiple Deprivation (IMD) score per MSOA in 2021. IMD is the official measure of relative socio-economic deprivation for small areas in England (10). We used IMD and its domains, which are composites of multiple risk factors for the wider determinants of health (11). | LSOA | DLUHC(12) |
|  | 2011 | Rural Urban Classification | Rural vs Urban Area, mapped to 2001 area codes. Rural areas are the reference group. | LSOA | DEFRA and ONS (13) |

*Table S2 Norfolk and Waveney MSOAs with 15 lowest and highest HLE at age 65 estimates for men in 2021, and risk factor prevalence by MSOA.*

| Lower tier local authority | MSOA name | Rural/urban classification^1^ | Life expectancy at birth, men (years) | Healthy life expectancy at birth, men (years) | Life expectancy at birth, women (years) | Healthy life expectancy at birth, women (years) | Life expectancy at 65 years, men (years) | Healthy life expectancy at 65 years, men (years) | Life expectancy at 65 years, women (years) | Healthy life expectancy at 65 years, women (years) | Alcohol admissions (rate) | Falls admissions, men (rate) | Falls admissions, women (rate) | Not eating 5-a-day (%) | Older person living alone (%) | Particulate matter 2.5 (μg per metre cubed) | Physical inactivity (%) | Road casualties (n) | IMD score | Income, net after housing (weekly, GBP) |
| --- | --- | --- | --- | --- | --- | --- | --- | --- | --- | --- | --- | --- | --- | --- | --- | --- | --- | --- | --- | --- |
| Great Yarmouth | Yarmouth Parade | U | 71 | 52 | 79 | 56 | 14 | 6 | 16 | 7 | 224 | 695 | 1245 | 50 | 61 | 7 | 29 | 9 | 67 | 390 |
| King's Lynn and West Norfolk | North Lynn | U | 78 | 59 | 78 | 58 | 16 | 7 | 13 | 5 | 187 | 1676 | 2286 | 43 | 54 | 6 | 31 | 7 | 47 | 425 |
| East Suffolk | Lowestoft Central | U | 73 | 54 | 79 | 58 | 16 | 7 | 18 | 9 | 109 | 1070 | 1425 | 35 | 57 | 8 | 20 | 0 | 47 | 462 |
| Great Yarmouth | Yarmouth Central & Northgate | U | 73 | 55 | 84 | 61 | 15 | 7 | 19 | 9 | 157 | 695 | 1245 | 50 | 57 | 6 | 29 | 4 | 56 | 433 |
| East Suffolk | Pakefield North | U | 77 | 61 | 82 | 61 | 14 | 7 | 19 | 10 | 102 | 1070 | 1425 | 35 | 52 | 7 | 20 | 0 | 33 | 475 |
| Great Yarmouth | Southtown & Cobholm | U | 72 | 56 | 82 | 60 | 14 | 7 | 20 | 10 | 129 | 695 | 1245 | 50 | 54 | 7 | 29 | 9 | 43 | 477 |
| Norwich | Bowthorpe & West Earlham | U | 78 | 61 | 83 | 63 | 14 | 7 | 20 | 10 | 123 | 1651 | 2093 | 48 | 52 | 7 | 19 | 13 | 34 | 438 |
| East Suffolk | Gunton West | U | 80 | 61 | 79 | 58 | 17 | 8 | 18 | 8 | 77 | 1070 | 1425 | 35 | 53 | 8 | 20 | 0 | 46 | 448 |
| Great Yarmouth | Gorleston West | U | 81 | 63 | 75 | 57 | 16 | 8 | 17 | 8 | 112 | 695 | 1245 | 50 | 50 | 7 | 29 | 6 | 37 | 469 |
| Great Yarmouth | Gorleston North | U | 78 | 59 | 81 | 59 | 15 | 8 | 18 | 9 | 105 | 695 | 1245 | 50 | 54 | 7 | 29 | 5 | 43 | 454 |
| King's Lynn and West Norfolk | Town, South Lynn & West Lynn | U | 78 | 61 | 81 | 62 | 16 | 8 | 20 | 10 | 190 | 1676 | 2286 | 43 | 65 | 6 | 31 | 5 | 37 | 446 |
| Norwich | Earlham | U | 77 | 59 | 81 | 60 | 16 | 8 | 20 | 10 | 118 | 1651 | 2093 | 48 | 51 | 7 | 19 | 12 | 35 | 465 |
| Norwich | Heartsease & Pilling Park | U | 76 | 59 | 81 | 62 | 15 | 8 | 18 | 10 | 123 | 1651 | 2093 | 48 | 53 | 7 | 19 | 12 | 35 | 469 |
| Norwich | Heigham Grove & St Augustines | U | 77 | 56 | 86 | 60 | 19 | 8 | 23 | 10 | 202 | 1651 | 2093 | 48 | 76 | 8 | 19 | 1 | 43 | 500 |
| Norwich | Mile Cross | U | 75 | 57 | 88 | 62 | 19 | 8 | 23 | 10 | 156 | 1651 | 2093 | 48 | 56 | 7 | 19 | 12 | 42 | 458 |
|  | | | | | | | | | | | | | | | | | | | | |
| North Norfolk | Beeston Regis, Saxthorpe & Aldborough | R | 86 | 71 | 86 | 70 | 21 | 13 | 21 | 13 | 72 | 1182 | 1779 | 39 | 39 | 6 | 16 | 8 | 21 | 585 |
| South Norfolk | Hingham, Morley & Barnham Broom | R | 83 | 70 | 87 | 70 | 21 | 13 | 22 | 13 | 73 | 1112 | 1833 | 39 | 40 | 6 | 19 | 5 | 15 | 612 |
| South Norfolk | Long Stratton, Carlton Rode & Tibenham | R | 83 | 69 | 86 | 70 | 21 | 13 | 23 | 13 | 88 | 1112 | 1833 | 39 | 38 | 6 | 19 | 8 | 15 | 590 |
| King's Lynn and West Norfolk | Dersingham, Sandringham & Massingham | R | 81 | 67 | 87 | 69 | 22 | 13 | 24 | 14 | 118 | 1676 | 2286 | 43 | 44 | 6 | 31 | 4 | 16 | 558 |
| North Norfolk | Holt & Weybourne | R | 86 | 69 | 88 | 70 | 21 | 13 | 23 | 14 | 89 | 1182 | 1779 | 39 | 48 | 6 | 16 | 3 | 18 | 571 |
| North Norfolk | Walsingham & Raynham | R | 87 | 70 | 90 | 70 | 22 | 13 | 25 | 14 | 105 | 1182 | 1779 | 39 | 43 | 6 | 16 | 7 | 25 | 542 |
| South Norfolk | Cringleford, Little Melton & Easton | R | 85 | 73 | 85 | 72 | 20 | 13 | 22 | 14 | 87 | 1112 | 1833 | 39 | 37 | 6 | 19 | 2 | 11 | 688 |
| Broadland | Spixworth & St Faiths | R | 89 | 72 | 91 | 74 | 24 | 13 | 26 | 15 | 81 | 1419 | 1531 | 39 | 42 | 6 | 22 | 4 | 10 | 554 |
| South Norfolk | Trowse, Poringland & Stoke Holy Cross | R | 83 | 71 | 88 | 73 | 19 | 13 | 24 | 15 | 79 | 1112 | 1833 | 39 | 42 | 6 | 19 | 4 | 8 | 654 |
| South Norfolk | Loddon, Surlingham & Alpington | R | 87 | 72 | 85 | 71 | 22 | 14 | 20 | 12 | 78 | 1112 | 1833 | 39 | 41 | 6 | 19 | 8 | 14 | 612 |
| East Suffolk | Southwold, Reydon & Wrentham | R | 86 | 71 | 87 | 71 | 21 | 14 | 25 | 15 | 71 | 1070 | 1425 | 35 | 46 | 6 | 20 | 0 | 17 | 602 |
| King's Lynn and West Norfolk | Wootton | U | 86 | 73 | 88 | 74 | 21 | 14 | 23 | 15 | 95 | 1676 | 2286 | 43 | 36 | 6 | 31 | 4 | 8 | 673 |
| North Norfolk | Wells & Blakeney | R | 87 | 72 | 89 | 72 | 22 | 14 | 24 | 15 | 100 | 1182 | 1779 | 39 | 46 | 6 | 16 | 2 | 22 | 613 |
| Norwich | Eaton | U | 85 | 73 | 90 | 73 | 20 | 14 | 25 | 15 | 81 | 1651 | 2093 | 48 | 47 | 7 | 19 | 2 | 8 | 669 |
| South Norfolk | Thurlton, Haddiscoe & Geldeston | R | 89 | 73 | 90 | 72 | 27 | 16 | 25 | 15 | 62 | 1112 | 1833 | 39 | 35 | 6 | 19 | 5 | 20 | 587 |

*Ordered according to HLE at 65 years for men (lowest to highest number of years). Risk factors are coloured according to deciles (or ranks where data is only available at local authority level), with 1 being the worst, coloured red, and 10 (8 for ranked data) being the best, coloured blue. Deciles 1 and 10 for HLE are also coloured red and blue respectively.*

*Notes: ^1^U= Urban, R = Rural.*

*Table S3 Norfolk and Waveney MSOAs with HLE estimates at birth and age 65 for men and women in 2021, and risk factor prevalence by MSOA*

| Lower tier local authority | MSOA name | Rural/urban classification^1^ | Life expectancy at birth, men (years) | Healthy life expectancy at birth, men (years) | Life expectancy at birth, women (years) | Healthy life expectancy at birth, women (years) | Life expectancy at 65 years, men (years) | Healthy life expectancy at 65 years, men (years) | Life expectancy at 65 years, women (years) | Healthy life expectancy at 65 years, women (years) | Alcohol admissions (rate) | Falls admissions, men (rate) | Falls admissions, women (rate) | Not eating 5-a-day (%) | Older person living alone (%) | Particulate matter 2.5 (μg per metre cubed) | Physical inactivity (%) | Road casualties (n) | IMD score | Income, net after housing (weekly, GBP) |
| --- | --- | --- | --- | --- | --- | --- | --- | --- | --- | --- | --- | --- | --- | --- | --- | --- | --- | --- | --- | --- |
| Great Yarmouth | Yarmouth Parade | U | 71 | 52 | 79 | 56 | 14 | 6 | 16 | 7 | 224 | 695 | 1245 | 50 | 61 | 7 | 29 | 9 | 67 | 390 |
| King's Lynn and West Norfolk | North Lynn | U | 78 | 59 | 78 | 58 | 16 | 7 | 13 | 5 | 187 | 1676 | 2286 | 43 | 54 | 6 | 31 | 7 | 47 | 425 |
| East Suffolk | Lowestoft Central | U | 73 | 54 | 79 | 58 | 16 | 7 | 18 | 9 | 109 | 1070 | 1425 | 35 | 57 | 8 | 20 | 0 | 47 | 462 |
| Great Yarmouth | Yarmouth Central & Northgate | U | 73 | 55 | 84 | 61 | 15 | 7 | 19 | 9 | 157 | 695 | 1245 | 50 | 57 | 6 | 29 | 4 | 56 | 433 |
| East Suffolk | Pakefield North | U | 77 | 61 | 82 | 61 | 14 | 7 | 19 | 10 | 102 | 1070 | 1425 | 35 | 52 | 7 | 20 | 0 | 33 | 475 |
| Great Yarmouth | Southtown & Cobholm | U | 72 | 56 | 82 | 60 | 14 | 7 | 20 | 10 | 129 | 695 | 1245 | 50 | 54 | 7 | 29 | 9 | 43 | 477 |
| Norwich | Bowthorpe & West Earlham | U | 78 | 61 | 83 | 63 | 14 | 7 | 20 | 10 | 123 | 1651 | 2093 | 48 | 52 | 7 | 19 | 13 | 34 | 438 |
| East Suffolk | Gunton West | U | 80 | 61 | 79 | 58 | 17 | 8 | 18 | 8 | 77 | 1070 | 1425 | 35 | 53 | 8 | 20 | 0 | 46 | 448 |
| Great Yarmouth | Gorleston West | U | 81 | 63 | 75 | 57 | 16 | 8 | 17 | 8 | 112 | 695 | 1245 | 50 | 50 | 7 | 29 | 6 | 37 | 469 |
| Great Yarmouth | Gorleston North | U | 78 | 59 | 81 | 59 | 15 | 8 | 18 | 9 | 105 | 695 | 1245 | 50 | 54 | 7 | 29 | 5 | 43 | 454 |
| King's Lynn and West Norfolk | Town, South Lynn & West Lynn | U | 78 | 61 | 81 | 62 | 16 | 8 | 20 | 10 | 190 | 1676 | 2286 | 43 | 65 | 6 | 31 | 5 | 37 | 446 |
| Norwich | Earlham | U | 77 | 59 | 81 | 60 | 16 | 8 | 20 | 10 | 118 | 1651 | 2093 | 48 | 51 | 7 | 19 | 12 | 35 | 465 |
| Norwich | Heartsease & Pilling Park | U | 76 | 59 | 81 | 62 | 15 | 8 | 18 | 10 | 123 | 1651 | 2093 | 48 | 53 | 7 | 19 | 12 | 35 | 469 |
| Norwich | Heigham Grove & St Augustines | U | 77 | 56 | 86 | 60 | 19 | 8 | 23 | 10 | 202 | 1651 | 2093 | 48 | 76 | 8 | 19 | 1 | 43 | 500 |
| Norwich | Mile Cross | U | 75 | 57 | 88 | 62 | 19 | 8 | 23 | 10 | 156 | 1651 | 2093 | 48 | 56 | 7 | 19 | 12 | 42 | 458 |
| Great Yarmouth | Yarmouth North | U | 80 | 61 | 83 | 62 | 17 | 9 | 18 | 8 | 125 | 695 | 1245 | 50 | 51 | 6 | 29 | 5 | 39 | 456 |
| East Suffolk | Lowestoft Harbour & Kirkley | U | 77 | 57 | 76 | 56 | 17 | 9 | 17 | 9 | 148 | 1070 | 1425 | 35 | 66 | 7 | 20 | 0 | 54 | 458 |
| King's Lynn and West Norfolk | Gaywood Chase & Old Gaywood | U | 79 | 64 | 79 | 62 | 16 | 9 | 19 | 10 | 199 | 1676 | 2286 | 43 | 51 | 7 | 31 | 1 | 27 | 475 |
| King's Lynn and West Norfolk | Marshland, Walpole & Walton | R | 81 | 65 | 84 | 66 | 17 | 9 | 21 | 11 | 129 | 1676 | 2286 | 43 | 40 | 6 | 31 | 9 | 29 | 535 |
| Norwich | Lakenham & Tuckswood | U | 80 | 62 | 87 | 64 | 17 | 9 | 22 | 11 | 155 | 1651 | 2093 | 48 | 60 | 7 | 19 | 6 | 34 | 483 |
| Breckland | Thetford North | U | 80 | 61 | 92 | 65 | 18 | 9 | 27 | 12 | 100 | 1446 | 2175 | 43 | 50 | 6 | 22 | 2 | 32 | 479 |
| Norwich | Catton Grove & Airport | U | 79 | 62 | 84 | 64 | 18 | 9 | 23 | 12 | 140 | 1651 | 2093 | 48 | 53 | 7 | 19 | 5 | 30 | 487 |
| King's Lynn and West Norfolk | Gaywood North Bank | U | 79 | 66 | 86 | 69 | 17 | 9 | 23 | 13 | 142 | 1676 | 2286 | 43 | 45 | 6 | 31 | 6 | 16 | 533 |
| Norwich | City Centre East | U | 83 | 65 | 83 | 65 | 18 | 10 | 18 | 10 | 126 | 1651 | 2093 | 48 | 71 | 7 | 19 | 4 | 25 | 550 |
| Great Yarmouth | Belton | R | 82 | 66 | 82 | 66 | 17 | 10 | 20 | 11 | 88 | 695 | 1245 | 50 | 41 | 6 | 29 | 3 | 21 | 521 |
| Great Yarmouth | Bradwell South & Hopton | U | 82 | 66 | 85 | 67 | 19 | 10 | 20 | 11 | 77 | 695 | 1245 | 50 | 48 | 6 | 29 | 1 | 18 | 573 |
| Great Yarmouth | Hemsby & Ormesby | R | 81 | 67 | 82 | 66 | 17 | 10 | 20 | 11 | 84 | 695 | 1245 | 50 | 38 | 6 | 29 | 5 | 22 | 558 |
| King's Lynn and West Norfolk | Fairstead & Springwood | U | 81 | 65 | 79 | 62 | 18 | 10 | 19 | 11 | 130 | 1676 | 2286 | 43 | 50 | 6 | 31 | 7 | 25 | 494 |
| King's Lynn and West Norfolk | Terrington & Clenchwarton | R | 83 | 66 | 83 | 65 | 20 | 10 | 21 | 11 | 87 | 1676 | 2286 | 43 | 39 | 6 | 31 | 10 | 24 | 525 |
| King's Lynn and West Norfolk | Upwell, Delph & Emneth | R | 83 | 66 | 84 | 66 | 19 | 10 | 21 | 11 | 96 | 1676 | 2286 | 43 | 40 | 6 | 31 | 12 | 29 | 508 |
| Breckland | Dereham West, Necton & Gressenhall | R | 82 | 67 | 83 | 68 | 17 | 10 | 21 | 12 | 88 | 1446 | 2175 | 43 | 38 | 6 | 22 | 5 | 17 | 581 |
| Breckland | Watton | R | 81 | 66 | 87 | 68 | 18 | 10 | 22 | 12 | 108 | 1446 | 2175 | 43 | 43 | 6 | 22 | 10 | 19 | 521 |
| Broadland | Hellesdon North West | U | 83 | 67 | 86 | 68 | 18 | 10 | 21 | 12 | 99 | 1419 | 1531 | 39 | 46 | 7 | 22 | 3 | 13 | 550 |
| King's Lynn and West Norfolk | Watlington, Wiggenhall & Terrington St John | R | 81 | 66 | 83 | 66 | 18 | 10 | 20 | 12 | 100 | 1676 | 2286 | 43 | 39 | 6 | 31 | 4 | 24 | 556 |
| North Norfolk | Cromer | R | 80 | 63 | 85 | 67 | 17 | 10 | 20 | 12 | 148 | 1182 | 1779 | 39 | 51 | 6 | 16 | 2 | 27 | 519 |
| Breckland | Attleborough | U | 82 | 67 | 86 | 69 | 17 | 10 | 23 | 13 | 99 | 1446 | 2175 | 43 | 50 | 6 | 22 | 4 | 15 | 556 |
| East Suffolk | Beccles | U | 82 | 66 | 84 | 66 | 17 | 10 | 22 | 13 | 100 | 1070 | 1425 | 35 | 48 | 7 | 20 | 0 | 24 | 531 |
| Norwich | Town Close | U | 78 | 64 | 86 | 69 | 17 | 10 | 21 | 13 | 106 | 1651 | 2093 | 48 | 64 | 7 | 19 | 15 | 18 | 592 |
| Norwich | City Centre West | U | 77 | 57 | 85 | 66 | 18 | 10 | 23 | 14 | 202 | 1651 | 2093 | 48 | 70 | 8 | 19 | 2 | 43 | 500 |
| Great Yarmouth | Gorleston South & Beach | U | 81 | 66 | 88 | 69 | 18 | 10 | 25 | 15 | 85 | 695 | 1245 | 50 | 46 | 7 | 29 | 7 | 24 | 560 |
| Breckland | Mundford, Weeting & Forest | R | 85 | 68 | 83 | 67 | 20 | 11 | 18 | 10 | 75 | 1446 | 2175 | 43 | 37 | 6 | 22 | 2 | 20 | 567 |
| East Suffolk | Normanston & Oulton Broad East | U | 82 | 65 | 84 | 65 | 21 | 11 | 19 | 10 | 80 | 1070 | 1425 | 35 | 48 | 8 | 20 | 0 | 26 | 508 |
| Norwich | University & Avenues | U | 85 | 65 | 84 | 64 | 20 | 11 | 19 | 10 | 110 | 1651 | 2093 | 48 | 55 | 7 | 19 | 4 | 21 | 458 |
| Great Yarmouth | Caister on Sea | R | 85 | 68 | 83 | 66 | 20 | 11 | 20 | 11 | 73 | 695 | 1245 | 50 | 46 | 6 | 29 | 4 | 21 | 540 |
| Norwich | New Catton & Mousehold North | U | 82 | 64 | 86 | 66 | 22 | 11 | 21 | 11 | 113 | 1651 | 2093 | 48 | 57 | 8 | 19 | 8 | 25 | 535 |
| Breckland | Dereham Central & Toftwood | U | 80 | 65 | 85 | 66 | 19 | 11 | 22 | 12 | 97 | 1446 | 2175 | 43 | 46 | 6 | 22 | 14 | 20 | 521 |
| Breckland | Wayland, Ellingham & Great Hockham | R | 80 | 65 | 84 | 69 | 18 | 11 | 19 | 12 | 102 | 1446 | 2175 | 43 | 32 | 6 | 22 | 7 | 24 | 588 |
| Broadland | Hellesdon South East | U | 84 | 70 | 85 | 69 | 19 | 11 | 20 | 12 | 84 | 1419 | 1531 | 39 | 45 | 7 | 22 | 4 | 11 | 556 |
| Broadland | Horsford, Hainford & Hevingham | R | 83 | 70 | 84 | 70 | 18 | 11 | 19 | 12 | 79 | 1419 | 1531 | 39 | 38 | 6 | 22 | 8 | 13 | 590 |
| East Suffolk | Carlton Colville | U | 82 | 67 | 85 | 67 | 19 | 11 | 22 | 12 | 77 | 1070 | 1425 | 35 | 45 | 7 | 20 | 0 | 18 | 552 |
| East Suffolk | Halesworth & Wangford | R | 81 | 66 | 80 | 65 | 17 | 11 | 21 | 12 | 77 | 1070 | 1425 | 35 | 49 | 6 | 20 | 0 | 19 | 550 |
| Great Yarmouth | Bradwell North | U | 85 | 70 | 86 | 70 | 20 | 11 | 21 | 12 | 78 | 695 | 1245 | 50 | 41 | 6 | 29 | 2 | 13 | 556 |
| Great Yarmouth | Fleggburgh, Rollesby & Martham | R | 84 | 67 | 84 | 68 | 21 | 11 | 21 | 12 | 67 | 695 | 1245 | 50 | 36 | 6 | 29 | 10 | 21 | 567 |
| King's Lynn and West Norfolk | Heacham & Snettisham | R | 84 | 69 | 84 | 68 | 19 | 11 | 21 | 12 | 112 | 1676 | 2286 | 43 | 43 | 6 | 31 | 7 | 19 | 550 |
| King's Lynn and West Norfolk | Southery, Feltwell & Hockwold | R | 82 | 67 | 88 | 69 | 19 | 11 | 23 | 12 | 78 | 1676 | 2286 | 43 | 40 | 6 | 31 | 3 | 21 | 529 |
| King's Lynn and West Norfolk | Stoke Ferry, Hilgay & Wimbotsham | R | 80 | 66 | 83 | 67 | 19 | 11 | 20 | 12 | 149 | 1676 | 2286 | 43 | 39 | 6 | 31 | 3 | 20 | 569 |
| North Norfolk | Fakenham | R | 83 | 67 | 85 | 67 | 18 | 11 | 20 | 12 | 115 | 1182 | 1779 | 39 | 49 | 6 | 16 | 10 | 19 | 523 |
| North Norfolk | North Walsham East | U | 79 | 64 | 85 | 67 | 20 | 11 | 21 | 12 | 112 | 1182 | 1779 | 39 | 50 | 6 | 16 | 3 | 20 | 538 |
| North Norfolk | North Walsham West | U | 77 | 62 | 85 | 67 | 20 | 11 | 21 | 12 | 112 | 1182 | 1779 | 39 | 49 | 6 | 16 | 8 | 20 | 538 |
| North Norfolk | Stalham & Sea Palling | R | 85 | 67 | 86 | 67 | 20 | 11 | 21 | 12 | 107 | 1182 | 1779 | 39 | 45 | 6 | 16 | 7 | 24 | 525 |
| South Norfolk | New Costessey | U | 83 | 67 | 84 | 67 | 20 | 11 | 21 | 12 | 111 | 1112 | 1833 | 39 | 46 | 6 | 19 | 7 | 13 | 594 |
| Breckland | Bawdeswell, Swanton Morley & North Elmham | R | 81 | 69 | 84 | 69 | 18 | 11 | 21 | 13 | 78 | 1446 | 2175 | 43 | 37 | 6 | 22 | 4 | 18 | 585 |
| Breckland | Swaffham | R | 83 | 66 | 85 | 66 | 18 | 11 | 23 | 13 | 109 | 1446 | 2175 | 43 | 45 | 6 | 22 | 12 | 25 | 519 |
| Breckland | Whissonsett, Litcham & Narborough | R | 81 | 67 | 86 | 70 | 19 | 11 | 21 | 13 | 92 | 1446 | 2175 | 43 | 37 | 6 | 22 | 11 | 19 | 573 |
| Broadland | Acle & Reedham | R | 82 | 68 | 86 | 69 | 20 | 11 | 21 | 13 | 97 | 1419 | 1531 | 39 | 46 | 6 | 22 | 4 | 15 | 608 |
| Broadland | Sprowston East | U | 79 | 67 | 85 | 69 | 19 | 11 | 23 | 13 | 81 | 1419 | 1531 | 39 | 44 | 7 | 22 | 5 | 8 | 581 |
| East Suffolk | Gunton East, Corton & Somerleyton | U | 83 | 69 | 88 | 71 | 18 | 11 | 23 | 13 | 72 | 1070 | 1425 | 35 | 38 | 6 | 20 | 0 | 17 | 594 |
| East Suffolk | Pakefield South & Kessingland | U | 84 | 68 | 86 | 67 | 19 | 11 | 23 | 13 | 78 | 1070 | 1425 | 35 | 45 | 6 | 20 | 0 | 20 | 558 |
| King's Lynn and West Norfolk | Downham Market | U | 78 | 65 | 84 | 66 | 18 | 11 | 21 | 13 | 103 | 1676 | 2286 | 43 | 50 | 6 | 31 | 5 | 19 | 554 |
| King's Lynn and West Norfolk | Grimston, Gayton & East Winch | R | 82 | 66 | 86 | 69 | 20 | 11 | 23 | 13 | 142 | 1676 | 2286 | 43 | 41 | 6 | 31 | 3 | 22 | 565 |
| South Norfolk | Diss & Roydon | U | 80 | 67 | 85 | 69 | 17 | 11 | 22 | 13 | 99 | 1112 | 1833 | 39 | 50 | 6 | 19 | 3 | 15 | 563 |
| South Norfolk | Hempnall, Ditchingham & Wortwell | R | 82 | 68 | 84 | 69 | 18 | 11 | 22 | 13 | 89 | 1112 | 1833 | 39 | 40 | 6 | 19 | 4 | 17 | 604 |
| South Norfolk | Hethersett | R | 80 | 67 | 89 | 71 | 18 | 11 | 24 | 13 | 98 | 1112 | 1833 | 39 | 43 | 6 | 19 | 5 | 10 | 640 |
| South Norfolk | Wymondham East & Spooner Row | U | 83 | 70 | 86 | 71 | 18 | 11 | 23 | 14 | 96 | 1112 | 1833 | 39 | 45 | 6 | 19 | 2 | 12 | 608 |
| North Norfolk | Sheringham | R | 83 | 68 | 88 | 71 | 18 | 11 | 23 | 15 | 105 | 1182 | 1779 | 39 | 45 | 6 | 16 | 5 | 15 | 621 |
| Breckland | Thetford South | U | 86 | 66 | 82 | 63 | 23 | 12 | 17 | 9 | 101 | 1446 | 2175 | 43 | 51 | 6 | 22 | 10 | 30 | 496 |
| King's Lynn and West Norfolk | Hunstanton | R | 84 | 65 | 81 | 64 | 22 | 12 | 19 | 11 | 125 | 1676 | 2286 | 43 | 48 | 6 | 31 | 2 | 27 | 544 |
| North Norfolk | Mundesley, Trunch & Bacton | R | 81 | 65 | 84 | 65 | 20 | 12 | 19 | 11 | 109 | 1182 | 1779 | 39 | 40 | 6 | 16 | 4 | 23 | 550 |
| Norwich | Earlham Road & College Road | U | 83 | 71 | 82 | 69 | 18 | 12 | 17 | 11 | 87 | 1651 | 2093 | 48 | 51 | 8 | 19 | 7 | 9 | 652 |
| Norwich | Thorpe Hamlet & Mousehold South | U | 86 | 68 | 86 | 66 | 21 | 12 | 21 | 11 | 141 | 1651 | 2093 | 48 | 61 | 8 | 19 | 5 | 28 | 569 |
| Breckland | Dereham North & Neatherd | U | 87 | 69 | 85 | 68 | 22 | 12 | 20 | 12 | 90 | 1446 | 2175 | 43 | 44 | 6 | 22 | 7 | 20 | 558 |
| Breckland | Thetford Central & East | U | 84 | 69 | 87 | 70 | 21 | 12 | 22 | 12 | 86 | 1446 | 2175 | 43 | 48 | 7 | 22 | 5 | 13 | 535 |
| South Norfolk | Scole, Dickleburgh & Bressingham | R | 82 | 69 | 86 | 70 | 19 | 12 | 21 | 12 | 87 | 1112 | 1833 | 39 | 39 | 6 | 19 | 3 | 18 | 606 |
| Breckland | Shipdham, Bradenham & Saham Toney | R | 82 | 67 | 87 | 69 | 19 | 12 | 22 | 13 | 103 | 1446 | 2175 | 43 | 40 | 6 | 22 | 9 | 18 | 542 |
| Broadland | Blofield, Lingwood & Upton | R | 85 | 71 | 86 | 70 | 20 | 12 | 21 | 13 | 85 | 1419 | 1531 | 39 | 39 | 6 | 22 | 9 | 11 | 629 |
| Broadland | Drayton & Thorpe Marriott | U | 85 | 71 | 83 | 69 | 20 | 12 | 22 | 13 | 110 | 1419 | 1531 | 39 | 42 | 6 | 22 | 6 | 10 | 606 |
| Broadland | Reepham, Cawston & Lenwade | R | 85 | 71 | 81 | 67 | 20 | 12 | 21 | 13 | 83 | 1419 | 1531 | 39 | 40 | 6 | 22 | 10 | 16 | 590 |
| Broadland | Sprowston Central | U | 85 | 70 | 87 | 71 | 20 | 12 | 22 | 13 | 105 | 1419 | 1531 | 39 | 43 | 7 | 22 | 4 | 13 | 563 |
| Broadland | Thorpe St Andrew South & Dussindale | U | 84 | 71 | 83 | 70 | 19 | 12 | 20 | 13 | 69 | 1419 | 1531 | 39 | 46 | 7 | 22 | 4 | 10 | 692 |
| Broadland | Wroxham, Rackheath & the Plumsteads | R | 86 | 71 | 85 | 71 | 21 | 12 | 20 | 13 | 100 | 1419 | 1531 | 39 | 37 | 6 | 22 | 3 | 12 | 648 |
| East Suffolk | Bungay & the Saints | R | 82 | 68 | 87 | 69 | 19 | 12 | 22 | 13 | 83 | 1070 | 1425 | 35 | 47 | 6 | 20 | 0 | 21 | 563 |
| King's Lynn and West Norfolk | West Winch, Marham & Shouldham | R | 86 | 71 | 88 | 70 | 21 | 12 | 23 | 13 | 95 | 1676 | 2286 | 43 | 37 | 6 | 31 | 5 | 16 | 573 |
| North Norfolk | Hoveton, Horning & Potter Heigham | R | 84 | 68 | 85 | 69 | 19 | 12 | 22 | 13 | 127 | 1182 | 1779 | 39 | 40 | 6 | 16 | 9 | 20 | 619 |
| North Norfolk | Melton Constable, Briston & Little Snoring | R | 82 | 67 | 88 | 70 | 20 | 12 | 23 | 13 | 74 | 1182 | 1779 | 39 | 41 | 6 | 16 | 7 | 19 | 583 |
| South Norfolk | Old Costessey & Queens Hills | U | 83 | 70 | 85 | 70 | 19 | 12 | 21 | 13 | 111 | 1112 | 1833 | 39 | 38 | 6 | 19 | 4 | 13 | 594 |
| Breckland | Buckenhams & Snetterton | R | 85 | 70 | 88 | 72 | 20 | 12 | 23 | 14 | 107 | 1446 | 2175 | 43 | 38 | 6 | 22 | 9 | 14 | 579 |
| Broadland | Aylsham | R | 80 | 67 | 88 | 71 | 19 | 12 | 23 | 14 | 115 | 1419 | 1531 | 39 | 45 | 6 | 22 | 17 | 14 | 621 |
| East Suffolk | Oulton Broad West | U | 83 | 71 | 86 | 71 | 18 | 12 | 23 | 14 | 66 | 1070 | 1425 | 35 | 37 | 7 | 20 | 0 | 10 | 602 |
| East Suffolk | Worlingham & Barnby | U | 85 | 70 | 87 | 71 | 20 | 12 | 22 | 14 | 56 | 1070 | 1425 | 35 | 40 | 7 | 20 | 0 | 13 | 608 |
| North Norfolk | Felmingham, Worstead & Happisburgh | R | 82 | 66 | 90 | 71 | 20 | 12 | 25 | 14 | 70 | 1182 | 1779 | 39 | 37 | 6 | 16 | 6 | 22 | 571 |
| South Norfolk | Harleston & Pulham | R | 85 | 70 | 87 | 70 | 20 | 12 | 22 | 14 | 90 | 1112 | 1833 | 39 | 44 | 6 | 19 | 7 | 14 | 552 |
| South Norfolk | Mulbarton, Tasburgh & Saxlingham Nethergate | R | 83 | 70 | 88 | 73 | 19 | 12 | 23 | 14 | 79 | 1112 | 1833 | 39 | 38 | 6 | 19 | 6 | 11 | 638 |
| South Norfolk | Wymondham West | U | 83 | 66 | 89 | 71 | 22 | 12 | 24 | 14 | 105 | 1112 | 1833 | 39 | 54 | 6 | 19 | 2 | 13 | 548 |
| Breckland | East Harling, Garboldisham & Kenninghall | R | 82 | 69 | 91 | 74 | 20 | 12 | 26 | 15 | 98 | 1446 | 2175 | 43 | 35 | 6 | 22 | 6 | 15 | 619 |
| Broadland | Brundall & Cantley | R | 82 | 69 | 90 | 73 | 19 | 12 | 25 | 15 | 98 | 1419 | 1531 | 39 | 40 | 6 | 22 | 7 | 12 | 637 |
| King's Lynn and West Norfolk | Brancaster, Burnham Market & Docking | R | 84 | 70 | 90 | 73 | 19 | 12 | 25 | 15 | 100 | 1676 | 2286 | 43 | 42 | 6 | 31 | 5 | 25 | 619 |
| North Norfolk | Overstrand, Roughton & the Runtons | R | 84 | 69 | 90 | 71 | 19 | 12 | 25 | 15 | 103 | 1182 | 1779 | 39 | 38 | 6 | 16 | 4 | 20 | 581 |
| Breckland | Hockering, Mattishall & Cranworth | R | 84 | 70 | 85 | 70 | 22 | 13 | 20 | 12 | 97 | 1446 | 2175 | 43 | 39 | 6 | 22 | 8 | 17 | 590 |
| Broadland | Thorpe St Andrew North | U | 84 | 69 | 87 | 70 | 21 | 13 | 22 | 12 | 93 | 1419 | 1531 | 39 | 48 | 7 | 22 | 4 | 12 | 563 |
| Broadland | Coltishall, Buxton & Frettenham | R | 83 | 69 | 85 | 71 | 21 | 13 | 20 | 13 | 79 | 1419 | 1531 | 39 | 40 | 6 | 22 | 5 | 14 | 588 |
| Broadland | Old Catton | U | 82 | 70 | 87 | 71 | 19 | 13 | 22 | 13 | 95 | 1419 | 1531 | 39 | 43 | 7 | 22 | 3 | 9 | 587 |
| Broadland | Taverham | U | 83 | 70 | 83 | 70 | 19 | 13 | 19 | 13 | 91 | 1419 | 1531 | 39 | 42 | 6 | 22 | 3 | 8 | 642 |
| East Suffolk | Oulton | U | 87 | 70 | 88 | 68 | 22 | 13 | 23 | 13 | 71 | 1070 | 1425 | 35 | 42 | 7 | 20 | 0 | 20 | 546 |
| North Norfolk | Beeston Regis, Saxthorpe & Aldborough | R | 86 | 71 | 86 | 70 | 21 | 13 | 21 | 13 | 72 | 1182 | 1779 | 39 | 39 | 6 | 16 | 8 | 21 | 585 |
| South Norfolk | Hingham, Morley & Barnham Broom | R | 83 | 70 | 87 | 70 | 21 | 13 | 22 | 13 | 73 | 1112 | 1833 | 39 | 40 | 6 | 19 | 5 | 15 | 612 |
| South Norfolk | Long Stratton, Carlton Rode & Tibenham | R | 83 | 69 | 86 | 70 | 21 | 13 | 23 | 13 | 88 | 1112 | 1833 | 39 | 38 | 6 | 19 | 8 | 15 | 590 |
| King's Lynn and West Norfolk | Dersingham, Sandringham & Massingham | R | 81 | 67 | 87 | 69 | 22 | 13 | 24 | 14 | 118 | 1676 | 2286 | 43 | 44 | 6 | 31 | 4 | 16 | 558 |
| North Norfolk | Holt & Weybourne | R | 86 | 69 | 88 | 70 | 21 | 13 | 23 | 14 | 89 | 1182 | 1779 | 39 | 48 | 6 | 16 | 3 | 18 | 571 |
| North Norfolk | Walsingham & Raynham | R | 87 | 70 | 90 | 70 | 22 | 13 | 25 | 14 | 105 | 1182 | 1779 | 39 | 43 | 6 | 16 | 7 | 25 | 542 |
| South Norfolk | Cringleford, Little Melton & Easton | R | 85 | 73 | 85 | 72 | 20 | 13 | 22 | 14 | 87 | 1112 | 1833 | 39 | 37 | 6 | 19 | 2 | 11 | 688 |
| Broadland | Spixworth & St Faiths | R | 89 | 72 | 91 | 74 | 24 | 13 | 26 | 15 | 81 | 1419 | 1531 | 39 | 42 | 6 | 22 | 4 | 10 | 554 |
| South Norfolk | Trowse, Poringland & Stoke Holy Cross | R | 83 | 71 | 88 | 73 | 19 | 13 | 24 | 15 | 79 | 1112 | 1833 | 39 | 42 | 6 | 19 | 4 | 8 | 654 |
| South Norfolk | Loddon, Surlingham & Alpington | R | 87 | 72 | 85 | 71 | 22 | 14 | 20 | 12 | 78 | 1112 | 1833 | 39 | 41 | 6 | 19 | 8 | 14 | 612 |
| East Suffolk | Southwold, Reydon & Wrentham | R | 86 | 71 | 87 | 71 | 21 | 14 | 25 | 15 | 71 | 1070 | 1425 | 35 | 46 | 6 | 20 | 0 | 17 | 602 |
| King's Lynn and West Norfolk | Wootton | U | 86 | 73 | 88 | 74 | 21 | 14 | 23 | 15 | 95 | 1676 | 2286 | 43 | 36 | 6 | 31 | 4 | 8 | 673 |
| North Norfolk | Wells & Blakeney | R | 87 | 72 | 89 | 72 | 22 | 14 | 24 | 15 | 100 | 1182 | 1779 | 39 | 46 | 6 | 16 | 2 | 22 | 613 |
| Norwich | Eaton | U | 85 | 73 | 90 | 73 | 20 | 14 | 25 | 15 | 81 | 1651 | 2093 | 48 | 47 | 7 | 19 | 2 | 8 | 669 |
| South Norfolk | Thurlton, Haddiscoe & Geldeston | R | 89 | 73 | 90 | 72 | 27 | 16 | 25 | 15 | 62 | 1112 | 1833 | 39 | 35 | 6 | 19 | 5 | 20 | 587 |

*Ordered according to HLE at 65 years for women (lowest to highest number of years). Risk factors are coloured according to deciles (or ranks where data is only available at local authority level), with 1 being the worst, coloured red, and 10 (8 for ranked data) being the best, coloured blue. Deciles 1 and 10 for HLE are also coloured red and blue respectively.*

*Notes: ^1^U= Urban, R = Rural.*

*Table S4 Pairwise Pearson correlation coefficients for outcomes and covariates included in linear regression models*

|  | HLE male at birth | HLE female at birth | HLE male at 65 | HLE female at 65 | Urban | % adults physically inactive | % not meeting 5 a day diet | Proportion age>66 living alone | Alcohol mortality male | Alcohol mortality female | Air pollution | Road casualty rate |
| --- | --- | --- | --- | --- | --- | --- | --- | --- | --- | --- | --- | --- |
|  |  |  |  |  |  |  |  |  |  |  |  |  |
| HLE male at birth | 1 |  |  |  |  |  |  |  |  |  |  |  |
| HLE female at birth | 0.86 | 1 |  |  |  |  |  |  |  |  |  |  |
| HLE male at 65 | 0.87 | 0.82 | 1 |  |  |  |  |  |  |  |  |  |
| HLE female at 65 | 0.71 | 0.89 | 0.74 | 1 |  |  |  |  |  |  |  |  |
| Urban | -0.41 | -0.43 | -0.42 | -0.39 | 1 |  |  |  |  |  |  |  |
| % adults physically inactive | -0.17 | -0.25 | -0.31 | -0.29 | 0.03 | 1 |  |  |  |  |  |  |
| % not meeting 5 a day diet | -0.39 | -0.35 | -0.42 | -0.38 | 0.19 | **0.48** | 1 |  |  |  |  |  |
| % age>66 living alone | -0.70 | -0.64 | -0.56 | **-0.50** | **0.59** | -0.05 | 0.33 | 1 |  |  |  |  |
| Alcohol mortality male | -0.48 | -0.46 | -0.47 | -0.43 | **0.41** | 0.14 | **0.79** | 0.51 | 1 |  |  |  |
| Alcohol mortality female | -0.46 | -0.45 | -0.45 | -0.40 | **0.43** | 0.00 | **0.59** | **0.48** | 0.91 | 1 |  |  |
| Air pollution | -0.42 | -0.44 | -0.40 | -0.37 | **0.70** | -0.13 | 0.16 | **0.62** | 0.53 | 0.56 | 1 |  |
| Road casualty rate | -0.10 | -0.04 | -0.11 | -0.08 | -0.18 | 0.06 | 0.37 | -0.06 | 0.10 | 0.02 | -0.14 | 1 |
| Falls admission rate | 0.05 | 0.08 | 0.06 | 0.07 | 0.00 | 0.12 | 0.06 | 0.10 | -0.22 | 0.03 | 0.26 | 0.05 |
| Weekly net income after housing | **0.83** | **0.86** | **0.78** | **0.76** | -0.39 | -0.25 | -0.36 | **-0.59** | -0.41 | -0.42 | -0.33 | -0.09 |

*Table S5 Variance inflation factors (VIF) for covariates included in linear regression models (Table 3)*

| **Outcome: HLE in females** | **VIF** |
| --- | --- |
| % Not meeting 5 a day diet | 3.63 |
| Alcohol mortality female | 3.08 |
| % age>66 living alone | 2.83 |
| Air pollution | 2.80 |
| Urban | 2.24 |
| % Adults physically inactive | 2.09 |
| Weekly net income after housing £ | 2.00 |
| Road casualty | 1.60 |
| Falls admission rate | 1.24 |
| Mean VIF | 2.39 |
|  |  |
| **Outcome: HLE in males** |  |
| **Covariate** | **VIF** |
| Alcohol mortality male | 8.86 |
| % Not meeting 5 a day diet | 8.64 |
| Air pollution | 3.54 |
| % age>66 living alone | 2.78 |
| Urban | 2.26 |
| % Adults physically inactive | 2.09 |
| Weekly net income after housing £ | 1.89 |
| Falls admission rate | 1.65 |
| Road casualty | 1.61 |
| Mean VIF | 3.70 |
|  |  |
|  |  |

**Supplementary Tables References**

1. Thangavel P, Park D, Lee Y-C. Recent insights into particulate matter (PM2. 5)-mediated toxicity in humans: an overview. International Journal of Environmental Research and Public Health. 2022;19(12):7511.

2. Department for Environment Food and Rural Affairs. Modelled background pollution data 2023 [Available from: <https://uk-air.defra.gov.uk/data/pcm-data>.]

3. Esri Inc. ArcGIS Pro 3.0.1. Redlands, CA: Environmental Systems Research Institute; 2022.

4. Lawson AB. Statistical methods in spatial epidemiology: John Wiley & Sons; 2013.

5. [dataset] Office for National Statistics. Lifestage of household reference person 2023 [Available from: <https://www.ons.gov.uk/datasets/create/filter-outputs/33976214-4b39-41eb-b567-97cc754cb468>.]

6. [dataset] Office for Health Improvement and Disparities. Fingertips: Public Health Profiles 2023 [Available from: <https://fingertips.phe.org.uk/>.]

7. [dataset] Department for Transport. Road Safety Data 2023 [Available from: <https://www.data.gov.uk/dataset/cb7ae6f0-4be6-4935-9277-47e5ce24a11f/road-safety-data>.]

8. [dataset] Department for Environment Food & Rural Affairs. Modelled background pollution data 2001–2022 [Available from: <https://uk-air.defra.gov.uk/data/pcm-data>.]

9. [dataset] Office for National Statistics. Income estimates for small areas, England and Wales 2020 [Available from: <https://www.ons.gov.uk/employmentandlabourmarket/peopleinwork/earningsandworkinghours/datasets/smallareaincomeestimatesformiddlelayersuperoutputareasenglandandwales>.]

10. Department for Levelling Up Housing and Communities. English Indices of Deprivation 2010/2019 [Available from: <https://www.gov.uk/government/collections/english-indices-of-deprivation>.]

11. Office for National Statistics. The 2011 Rural-Urban Classification For Small Area Geographies: A User Guide and Frequently Asked Questions (v1.0) 2013 [updated Aug 28. Available from: <https://assets.publishing.service.gov.uk/government/uploads/system/uploads/attachment_data/file/239478/RUC11user_guide_28_Aug.pdf>.]

12. [dataset] Ministry of Housing Communities & Local Government. English indices of deprivation 2019 2019 [Available from: <https://www.gov.uk/government/statistics/english-indices-of-deprivation-2019>.]

13. [dataset] Office for National Statistics. Rural/urban classifications 2011 [Available from: <https://www.ons.gov.uk/methodology/geography/geographicalproducts/ruralurbanclassifications>.]
