# Supplementary figures and images for: How do associations between healthy life expectancy and risk factors vary across small geographic areas in a UK integrated care system? Cross-sectional study
Source: BMJ Open. 2026 Jul 13;16(7):e108114. doi: 10.1136/bmjopen-2025-108114 (PMC13365761; doi:10.1136/bmjopen-2025-108114)

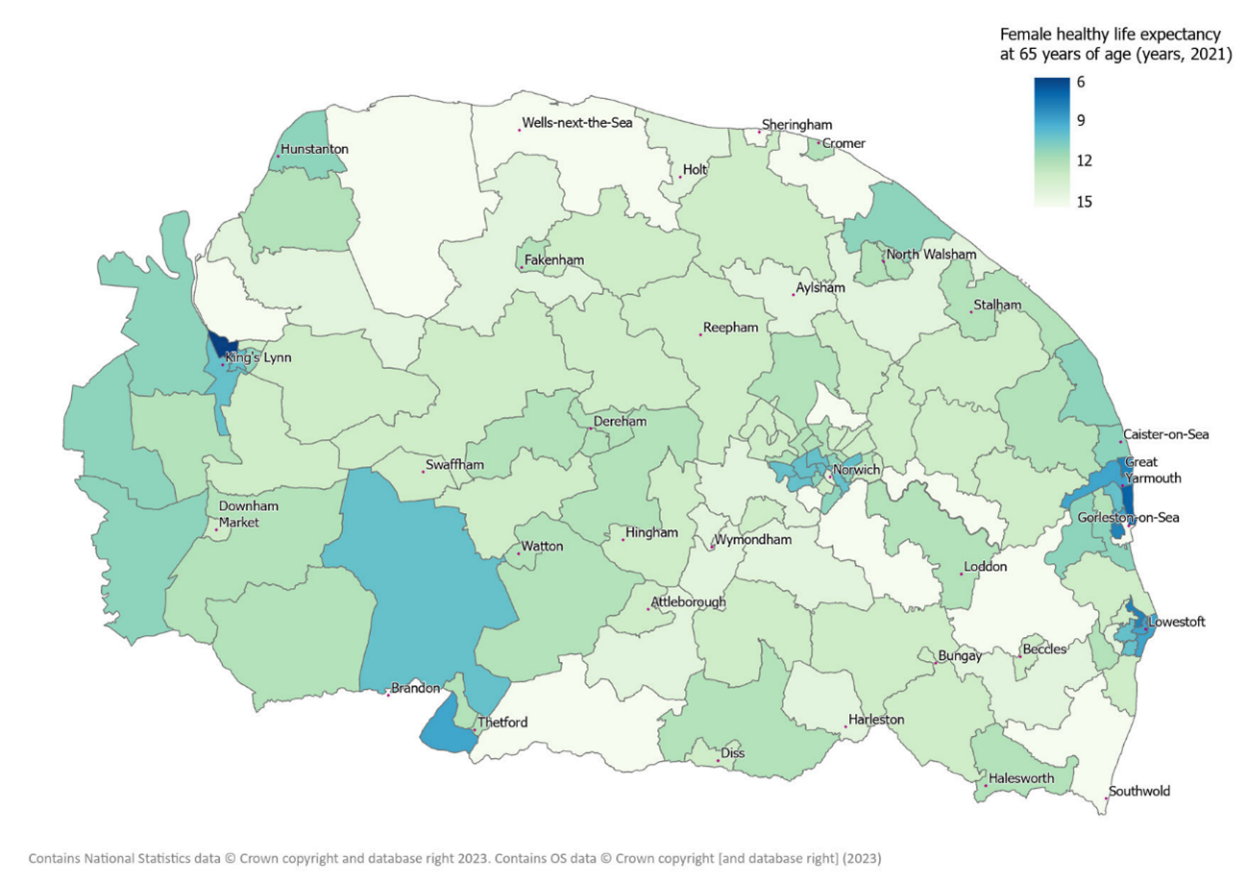

Supplement: online supplemental figure 1 [file bmjopen-16-7-s002.tif]

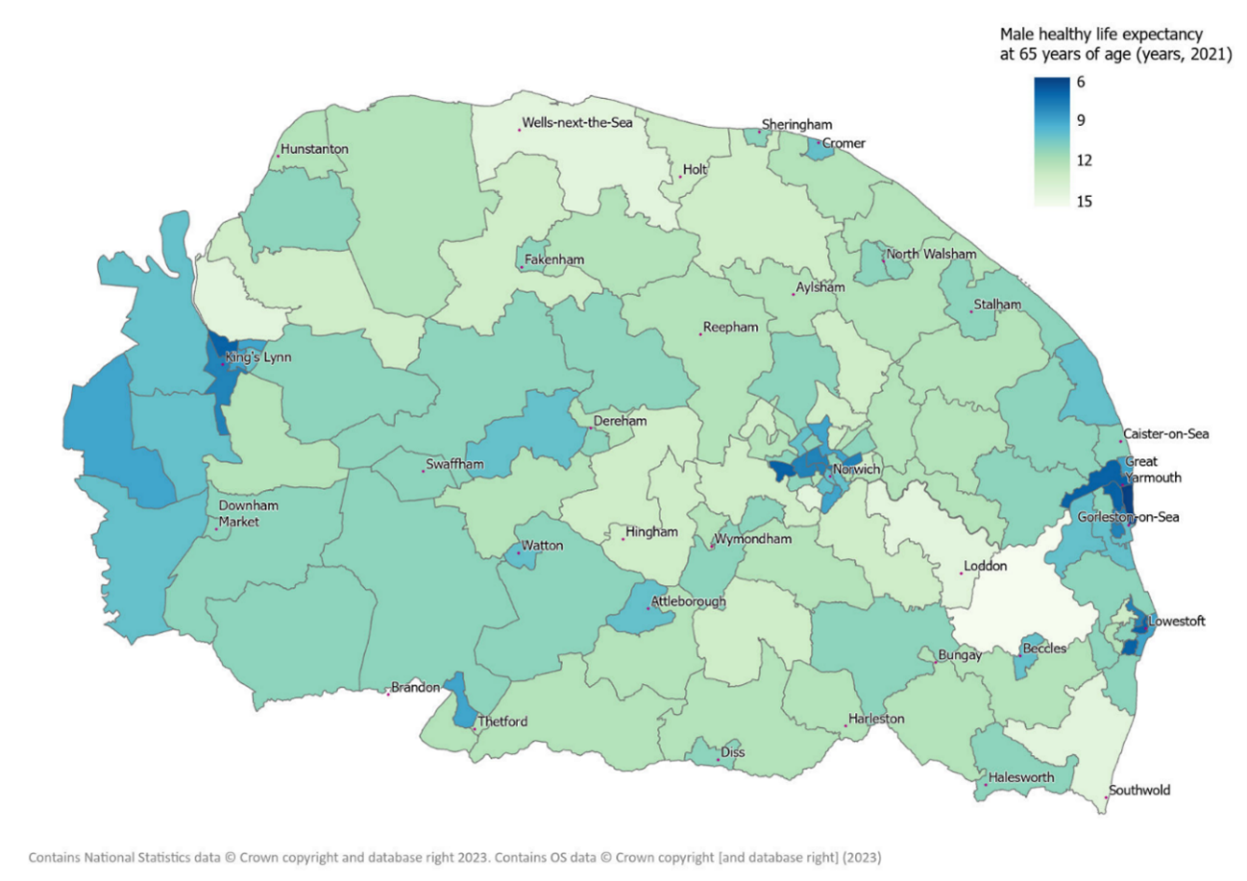

Supplement: online supplemental figure 2 [file bmjopen-16-7-s003.tif]
