# Supplementary material for: How do associations between healthy life expectancy and risk factors vary across small geographic areas in a UK integrated care system? Cross-sectional study
Source: BMJ Open. 2026 Jul 13;16(7):e108114. doi: 10.1136/bmjopen-2025-108114 (PMC13365761; doi:10.1136/bmjopen-2025-108114)
Supplement: Supplementary file 8 [file bmjopen-2025-108114.reviewer_comments.pdf]

PEER REVIEW HISTORY

BMJ Open publishes all reviews undertaken for accepted manuscripts. Reviewers are asked to complete a checklist review form and are provided with free text boxes to elaborate on their assessment. These free text comments are reproduced below.

ARTICLE DETAILS

Title (Provisional)

How do associations between healthy life expectancy and risk factors vary across small geographic areas in a UK integrated care system? Cross-sectional study

Authors

Bachmann, Max; Saunders, Mike; Shabuz, Zillur Rahman; Dalton, Alice; Enwo, Oby O; Brainard, Julii; Jones, Charlotte E L; Burke, Amanda; Onoja, Mary; Ponzo, Aisling; Howard Wilsher, Stephanie; Gentry, Sarah; Steel, Nicholas

---

VERSION 1 - REVIEW

**Reviewer**	**1**
**Name**	**Dack, Kyle**
**Affiliation**	**University of Bristol, MRC Integrative Epidemiology Unit**
**Date**	**07-Oct-2025**
**COI**	**None**

Review summary:

The study has high novelty and the introduction and discussion were well written. Unfortunately there are major issues in the statistical methodology which risk introducing bias. The analysis is ecological at the level of MSOA, but in parts of the paper results are implied to be at an individual-level. The outcome – HLE – is an estimate which contains uncertainty, but the modelling strategy treats it as a fixed quantity. In addition, the use of stepwise variable selection risks reporting bias, as variables with strong evidence- and theoretical-based rationale for inclusion were subsequently dropped.

Abstract:

1. The objective gives a rationale that local-specific statistics on life expectancy and risk are required. This is a reasonable rationale, but the title and abstract do not provide information on the location (e.g. East of England) which would hinder anyone searching for this type of information, and leave readers unsure what locale this study is relevant to. I suggest specifying on line 30 and/or 34.

2. Line 35: the “population” in this study are the MSOA – no individual-level data is used. This is an issue in various parts of the paper which has a high risk of confusing readers, and I recommend rephrasing throughout to ensure that it is clear that the results are at an MSOA level.

3. Lines 49-51. The 95% CI do not seem accurate – in some cases the lower limit is greater than the higher limit. Additionally, these appear to be coefficients for a continuous outcome, but it is unclear what this represents – i.e. what are the units of the model results? I think it may be years, but this should be reported here and in all parts of the results.

Introduction:

4. Clear rationale and background – no suggestions.

Methods:

5. There are several key sections required in the STROBE guidance which are currently absent and impair the transparency of the methods. I have given suggestions below but more detailed information can be found in the STROBE elaboration document.

6. Study design – key information is currently not reported, and this is typically contained in a section at the beginning of the methods which I suggest adding, e.g. that it is cross-sectional, the dates the data pertains to, and the primary analysis method. This would align with STROBE requirements (item 4)

7. Data source - The first paragraph describes the setting: the geographical area the study population was found in. However, the data source is not directly explained. I suggest adding a “data source” section including how it was accessed, key assumptions (i.e. the data includes everyone who responded?), and any inclusions or exclusions.

8. Line 112: The data is at the level of MSOA, as far as I can see there is not individual-level data. This is important to clarify because it impacts the understanding of the subsequent methods.

9. Line 118: The subsequent two paragraphs describe the outcome, and it would be clearer to readers if under an “outcome” subheading. The methodological details are well described but the units of the final estimate are not reported – years? Also, similar to the above, please clarify that HLE was estimated per-MSOA, not per-person.

I have two major statistical concerns:

10. The uncertainty in the outcome was not propagated into the model, which risks biased coefficients and deflated standard errors. The Sullivan method is an estimate of HLE which contain uncertainty, not fixed quantities. Modelling these as exact outcomes means this uncertainty is ignored, and this both deflates standard errors but also could bias the point estimate towards zero. Alternative models should be used which can incorporate uncertainty in the dependent variable. A couple of examples for how to achieve this are here: https://pubmed.ncbi.nlm.nih.gov/38146817/, https://pubmed.ncbi.nlm.nih.gov/23778148/

11. Although the authors identified evidence-based risk factors to evaluate against the outcome, this strength of the study was undermined by the use of stepwise elimination to remove variables based on significance. This overrides prior evidence, increases the risk of spurious associations and omits variables which could be necessary to minimize confounding bias. Given the goals of the paper are to inform clinical knowledge, it would be more suitable to select variables based on existing theory and to minimize confounding.

Results

12. Overall, the results would benefit greatly from clarifying the unit of analysis. The analysis was at an MSOA level but in some parts the labelling or description describe findings as if based on individual data. This could confuse readers, and risks implying individual-level inference from aggregate data. The tables (specifically table 1) and accompanying text should consistently present results as MSOA-level estimates.

13. Table 1 – these findings were not part of the statistical analysis in the methodology, so it is unclear how exactly they were calculated. I assume with weightings for each MSOA but to ensure an accurate interpretation this should be described in the methodology.

14. Table 2- Please add this coding to the methodology. The blue and red categorization are not consistency highest and lowest, sometimes red is the highest decile (e.g. falls admissions), but in others it is the lowest (e.g. income, life expectancy).

15. Line 223: Please add the effect estimates, ideally 95% CI, to align with STROBE reporting guidance.

16. An issue throughout the results is a focus on statistical significance, which risks reporting bias, as factors without any significant association are not reported. While it is not efficient to report every non significant finding, the other factors should at least be mentioned in brief, to ensure transparency and because null findings could be equally as important in the future.

Discussion

17. Generally very well written but due to the aforementioned issues I have low certainty in the model results. Line 286-287 seem to contradict the modelling strategy of using stepwise elimination, if there is good quality evidence linking all to health outcomes.

**Reviewer**	**2**
**Name**	**Wongrith , Paleeratana**
**Affiliation**	**Walailak University, School of Public Health**
**Date**	**18-Feb-2026**
**COI**	**None**

Overall assessment

Thanks to the editors for the opportunity to review this well-structured cross-sectional manuscript. This is a population study examining associations between healthy life expectancy (HLE) and area-level risk factors within one English Integrated Care System (ICS). The topic is policy-relevant and methodologically grounded in publicly available data sources. The manuscript adheres broadly to STROBE reporting standards and is clearly written. However, several methodological clarifications and analytical justifications are required to strengthen internal validity, interpretability, and reproducibility.

1. BACKGROUND / INTRODUCTION

Strengths

- The scientific rationale is clearly stated (Introduction paras 1–2).
- The policy relevance of estimating HLE at small-area level is well justified.
- The objective is explicitly defined (Introduction para 3).
- The study design is clearly indicated in the title and abstract as cross-sectional
- The abstract is generally balanced and informative

Issues / Points for Clarification

1. **Conceptual framework**
    - The introduction would benefit from a clearer conceptual model explaining the assumed pathway between area-level risk factors and HLE.
    - At present, risk factors appear selected based on availability and evidence, but their interrelationships (e.g., deprivation, income, behavioural risk clustering) are not explicitly theorised.
2. **Ecological inference**
    - The ecological nature of inference (MSOA-level data) should be stated explicitly earlier in the Introduction.
    - A brief acknowledgment of ecological fallacy would strengthen the framing.
3. **Justification for ICS focus**
    - The rationale for focusing on a single ICS is implied but could be strengthened by clarifying why this ICS is particularly informative (e.g., diversity, deprivation gradient, rural–urban mix).

2. METHODS

Strengths

- Study design is clearly stated (cross-sectional population-based study)
- Setting and population are clearly described (128 MSOAs, 1,032,500 people)
- Ethical approval is appropriately documented
- Data sources for risk factors are transparently described, including spatial methods for PM2.5
- Funding statement and conflicts of interest are clearly reported

Major Methodological Comments

1. Study size and statistical power

The manuscript states the study size (128 MSOAs), but there is:

- No formal discussion of statistical power.
- No consideration of model overfitting relative to number of predictors.

Given 10 candidate predictors and 128 area-level observations, please justify:

- Variable selection approach.
- Whether multicollinearity was assessed (e.g., VIF).
- How overfitting risk was mitigated.

Reference 24 suggests awareness of variable selection methodology but the operational strategy must be clearly described in Methods.

2. Handling of missing data

STROBE item 12c indicates missing data handling is discussed in

Discussion but:

- Missingness procedures should be described in Methods, not solely in Discussion.
- Please clarify:
    - Were there missing MSOAs?
    - Were complete-case analyses used?
    - Were any imputations performed?

3. Risk factor temporal alignment

Several datasets are not from 2021 (e.g., income 2019–20

This introduces:

- Potential temporal mismatch bias.
- Risk of misclassification if area conditions changed.

Please:

- Quantify time gaps for each exposure.
- Provide sensitivity analysis (if feasible).
- Justify why this does not materially affect conclusions.

4. Multicollinearity and deprivation

Index of Multiple Deprivation (IMD) and income are conceptually overlapping variables.

Please clarify:

- Were correlation matrices examined?
- Was IMD included alongside income in the same model?
- How was redundancy handled?

This is particularly important because deprivation indices may capture several of the other included risk factors.

5. Model specification clarity

The abstract reports regression coefficients (e.g., income coefficient 0.02)

but:

- The unit of change must be explicitly stated (e.g., per £1? per £100? per SD?).
- It is unclear whether predictors were standardised.
- Clarify whether coefficients are linear and whether assumptions (normality, homoscedasticity) were tested.

6. Ecological bias

As this is an ecological analysis at MSOA level:

- Individual-level causal interpretation must be avoided.
- Explicit statement on ecological fallacy should be included in Methods and Discussion

7. Sensitivity analyses

STROBE indicates no sensitivity analyses were performed
Given:

- Risk factor selection decisions,
- Potential collinearity,
- Temporal mismatch,

At least one sensitivity model (e.g., removing IMD or testing alternative model forms) would strengthen robustness.

3. RESULTS

Strengths

- Clear reporting of HLE ranges and area differences
- Regression coefficients reported with confidence intervals.

- Tables referenced appropriately, T1 about outcome variable in ecological outcome indicator.

Points Requiring Clarification

1. Interpretation of coefficient magnitude

The abstract reports: Income coefficient 0.02 (95% CI 0.01–

0.02) However:

- The practical meaning of 0.02 years per unit income is unclear.
- Consider translating into meaningful increments (e.g., per £1,000 weekly income increase).
- Consider presenting predicted HLE differences across deprivation quintiles.

2. Sex-stratified analysis

Results focus primarily on males aged 65 in the abstract.

Please clarify:

- Were models run separately for men and women?
- Why are female regression results not equally presented?
- Were interaction terms tested?

3. Multiple comparisons

With 10 candidate risk factors:

- Was adjustment for multiple testing considered?
- Clarify selection pathway to final model.

4. Spatial autocorrelation

Given MSOA-level data:

- Were spatial dependencies tested (e.g., Moran's I)?
- If spatial clustering exists, OLS assumptions may be violated.

Given spatial methods were used in exposure construction spatial dependence should be considered analytically.

4. DISCUSSION

Strengths

- Key findings summarised clearly.
- Strengths and limitations section included

- Funding independence stated

Areas for Strengthening

1. Causal language

Some phrasing implies directional impact (e.g., prioritise interventions based on associations

Given cross-sectional ecological design:

- Emphasise association rather than causation.
- Clarify that reverse causality cannot be excluded.

2. Magnitude of potential bias

STROBE recommends discussing direction and magnitude of

bias Expand discussion of:

- Measurement error in behavioural exposures (diet, physical inactivity).
- Ecological misclassification.
- Temporal mismatch bias.
- Unmeasured confounding (e.g., smoking).

3. External validity

Generalisability is briefly addressed.

Please clarify:

- Is this ICS representative of England?
- Would results differ in urban metropolitan ICS areas?

4. Policy implications

The manuscript appropriately highlights local planning utility.

However:

- Include caution regarding overinterpretation.
- Consider discussing cost-effectiveness uncertainty of proposed interventions.

Statistical Review Recommendation

Yes - this paper would benefit from specialist statistical review.

Key reasons:

- Variable selection methodology.
- Multicollinearity concerns.
- Spatial autocorrelation not addressed.
- Lack of sensitivity analyses.
- Ecological modelling assumptions.

Checklist-Specific Comments (Where "No" or "Partially")

Missing data handling (12c): Insufficiently described in

Methods. **Sensitivity analyses (12e):** Not conducted –

recommended.

Spatial dependency: Not addressed.

Model transparency: Units and scaling of predictors require clarification

Minor Comments

- Title: grammatical correction needed — "risk factor vary" → "risk factors vary"
- Ensure consistent reporting of decimal places.
- Clarify whether regression diagnostics were performed.
- Consider adding a flow diagram for clarity (optional but recommended by STROBE).

Conclusion

This is a strong, policy-relevant ecological study using publicly available data. The manuscript would be suitable for publication after **moderate revision**, primarily addressing:

1. Statistical model transparency and justification
2. Multicollinearity and spatial considerations
3. Missing data and sensitivity analyses
4. Clearer articulation of ecological limitations

Once strengthened analytically, this paper will provide valuable methodological guidance for local public health intelligence units.

VERSION 1 - AUTHOR RESPONSE

Reviewer: 1

Dr. Kyle Dack, University of Bristol

Comments to the Author:	Authors' response

Review summary:<br>The study has high novelty and the introduction and discussion were well written. Unfortunately there are major issues in the statistical methodology which risk introducing bias. The analysis is ecological at the level of MSOA, but in parts of the paper results are implied to be at an individual-level. The outcome – HLE – is an estimate which contains uncertainty, but the modelling strategy treats it as a fixed quantity. In addition, the use of stepwise variable selection risks reporting bias, as variables with strong evidence- and theoretical-based rationale for inclusion were subsequently dropped.	Thankyou for these helpful comments.<br><br>The analysis is at MSOA level and this has been clarified throughout the paper.<br><br>Thank you for this comment about HLE being an estimate which contains uncertainty and have reviewed the examples about alternative modelling strategies in the suggested papers. After careful consideration, we think that they are not relevant to this paper and that our method is more appropriate. We give more details about our rationale for this response in the table below (point 10).<br><br>We agree with this point about stepwise regression and have removed it from the analysis.
Abstract:<br>1. The objective gives a rationale that local-specific statistics on life expectancy and risk are required. This is a reasonable rationale, but the title and abstract do not provide information on the location (e.g. East of England) which would hinder anyone searching for this type of information, and leave readers unsure what locale this study is relevant to. I suggest specifying on line 30 and/or 34.	We have added the location to line 32, which now reads:<br>*'Setting: Norfolk and Waveney Integrated Care System.'*
2. Line 35: the "population" in this study are the MSOA – no individual-level data is used. This is an issue in various parts of the paper which has a high risk of confusing readers, and I recommend rephrasing throughout to ensure that it is clear that the results are at an MSOA level.	We have removed the number of people in line 33, which now reads:<br>*'Population: 128 Middle layer Super Output Areas and eight Lower Tier Local Authority Areas'.*<br>The analysis is at MSOA level and this has been clarified throughout the paper.
3. Lines 49-51. The 95% CI do not seem accurate – in some cases the lower limit is greater than the higher limit. Additionally, these appear to be coefficients for a continuous outcome, but it is unclear what this represents – i.e. what are the units of the model results? I think it may be years, but this should be reported here and in all parts of the results.	We have revised the results section of the abstract, and added the units. Lines 46-51 now read:<br>*'Higher income was strongly associated with all healthy life expectancies: £100 higher weekly income was associated with 4.4 (95% confidence limits 3.5,5.2) and 4.6 (3.8,5.4) years greater HLE at birth in males and females respectively, and with 1.7 (1.3,2.2) and 2.0 (1.5,2.5) greater HLE at age 65. Higher percentage of older adults living alone was associated with lower HLE at birth in males and*

	females. Physical inactivity was associated with lower HLE at 65 in males and at birth in females.'
Introduction:<br>4. Clear rationale and background – no suggestions.	Thank you
Methods:<br>5. There are several key sections required in the STROBE guidance which are currently absent and impair the transparency of the methods. I have given suggestions below but more detailed information can be found in the STROBE elaboration document.	Thank you. We have responded to the suggestions where they are made below.
6. Study design – key information is currently not reported, and this is typically contained in a section at the beginning of the methods which I suggest adding, e.g. that it is cross-sectional, the dates the data pertains to, and the primary analysis method. This would align with STROBE requirements (item 4)	We have added this information to the beginning of the methods section:<br>*'This cross-sectional study uses a standard approach to estimating healthy life expectancies in MSOAs in Norfolk and Waveney. Associations between HLEs and risk factors for poorer health and mortality were estimated using correlation coefficients and linear regression. Data pertain to 2017 to 2021.'* (lines 119-122).
7. Data source - The first paragraph describes the setting: the geographical area the study population was found in. However, the data source is not directly explained. I suggest adding a "data source" section including how it was accessed, key assumptions (i.e. the data includes everyone who responded?), and any inclusions or exclusions.	Full information on risk factors and data sources in included as supplementary material, due to the volume of information. This is referred to in lines 186-187:<br><br>'*Risk factors and data sources are described in the Supplementary material and Supplementary Table S1*.'
8. Line 112: The data is at the level of MSOA, as far as I can see there is not individual-level data. This is important to clarify because it impacts the understanding of the subsequent methods.	We have added this clarification in lines 119-120:<br>'*This cross-sectional study uses a standard approach to estimating healthy life expectancies in MSOAs in Norfolk and Waveney.'*
9. Line 118: The subsequent two paragraphs describe the outcome, and it would be clearer to readers if under an "outcome" subheading. The methodological details are well described but the units of the final estimate are not reported – years? Also, similar to the above, please clarify that HLE was estimated per-MSOA, not per-person.	We have added an 'outcome' subheading in line 129, above the subsequent 2 paragraphs.<br><br>We have added this clarification in line 137:<br>'*We estimated HLE at MSOA level and the units are years*.'

I have two major statistical concerns:<br>10. The uncertainty in the outcome was not propagated into the model, which risks biased coefficients and deflated standard errors. The Sullivan method is an estimate of HLE which contain uncertainty, not fixed quantities. Modelling these as exact outcomes means this uncertainty is ignored, and this both deflates standard errors but also could bias the point estimate towards zero. Alternative models should be used which can incorporate uncertainty in the dependent variable. A couple of examples for how to achieve this are here: https://pubmed.ncbi.nlm.nih.gov/38146817/, https://pubmed.ncbi.nlm.nih.gov/23778148/	Thankyou for raising this point. We have read the suggested examples in the papers and we think that they are not relevant to this paper because:<br>1. https://pubmed.ncbi.nlm.nih.gov/38146817/ provides a method for estimating HLEs for much smaller populations than our MSOA populations. In their introduction, the authors provide the rationale for their method by saying that HLEs cannot be calculated using life table methods (like we used) for populations smaller than 5000 person-years at risk, and where morbidity data is only available from smaller sample surveys. In our study all MSOAs had populations greater than 5000, and morbidity data are we available for the entire population of each MSOA because it was collected by census. Thus our method - following Public Health England guidance, which recommends minimum samples of 5000 person years per small area - is appropriate.<br>2. Neither of the examples provided describe how to account for uncertainty in HLE estimates for linear regression models, like ours, that have HLE as outcome variable. (https://pubmed.ncbi.nlm.nih.gov/23778148/ is about predicting future HLEs, not about multiple regression models.) Presumably it is possible to do this, but in our view it is unnecessary. In any epidemiological study there is uncertainty about measurements, but it is not usually necessary to account for the uncertainty about each observation in every statistical analysis. For example, there is invariably random error in blood pressure measurement in an individual, which is why the mean of several measurement is used for analysis. However it is not necessary to account for the uncertainty around the mean when analysing blood pressure data (even though it would be possible).
11. Although the authors identified evidence-based risk factors to evaluate against the outcome, this strength of the study was undermined by the use of	Thankyou, we agree with this point and have removed the stepwise elimination of variables.

stepwise elimination to remove variables based on significance. This overrides prior evidence, increases the risk of spurious associations and omits variables which could be necessary to minimize confounding bias. Given the goals of the paper are to inform clinical knowledge, it would be more suitable to select variables based on existing theory and to minimize confounding.	
Results<br>12. Overall, the results would benefit greatly from clarifying the unit of analysis. The analysis was at an MSOA level but in some parts the labelling or description describe findings as if based on individual data. This could confuse readers, and risks implying individual-level inference from aggregate data. The tables (specifically table 1) and accompanying text should consistently present results as MSOA-level estimates.	Thankyou for this point. The analysis is at MSOA level and this has now been clarified throughout the paper.<br>The title of Table 1 is:<br>'*Healthy life expectancy and life expectancy in Norfolk and Waveney MSOAs'*<br>Table 1 specifies that the units are years.
13. Table 1 – these findings were not part of the statistical analysis in the methodology, so it is unclear how exactly they were calculated. I assume with weightings for each MSOA but to ensure an accurate interpretation this should be described in the methodology.	We have added this second sentence to Statistical methods: '*We estimated the mean, standard deviation and range of each HLE outcome across all MSOAs.'* (lines 190-191)
14. Table 2- Please add this coding to the methodology. The blue and red categorization are not consistency highest and lowest, sometimes red is the highest decile (e.g. falls admissions), but in others it is the lowest (e.g. income, life expectancy).	Thankyou for pointing this out. The colour coding in table 2 is intended as a visual aid to interpretation of a data-dense table. We describe it in the text as follows:<br>'*To aid identification of clusters of risk factors and low HLE, these tables are coloured by decile with red for short HLE and high risk, and blue for long HLE and lower risk*.' (lines 231-233)<br>We have changed the footnote to clarify the meaning of the blue and red colour coding as follows:<br>*'HLE estimates in the worst decile are highlighted in red, and those in the best decile in blue. Risk factor estimates in the worst decile are highlighted in red and those in the best decile in blue.'*<br>For example, high falls are red as they reflect a poor health outcome, whereas low income and

	low life expectancy are also red as they are also indicators of poor health.
15. Line 223: Please add the effect estimates, ideally 95% CI, to align with STROBE reporting guidance.	We have revised this section of the results and included 95% CI (lines 277-280):<br>*'In linear regression models (Tables 3 (females) and 4 (males)), higher income was strongly associated with all HLEs: £100 higher weekly income was associated with 4.4 (95% confidence limits 3.5,5.2) and 4.6 (3.8,5.4) years greater HLE at birth in males and females, respectively, and with 1.7 (1.3,2.2) and 2.0 (1.5,2.5) greater HLE at age 65.'*
16. An issue throughout the results is a focus on statistical significance, which risks reporting bias, as factors without any significant association are not reported. While it is not efficient to report every non significant finding, the other factors should at least be mentioned in brief, to ensure transparency and because null findings could be equally as important in the future.	Thankyou for this suggestion. The regression tables report the non-significant associations, and we have added this brief summary of the most important ones in lines 298-300:<br>'*Some risk factors showed no statistically significant association with HLE, notably urban vs rural location, percentage of adults not meeting the five a day dietary target, and air pollution.*'
Discussion<br>17. Generally very well written but due to the aforementioned issues I have low certainty in the model results. Line 286-287 seem to contradict the modelling strategy of using stepwise elimination, if there is good quality evidence linking all to health outcomes.	We have revised the methods as described in point 11 above and are no longer using stepwise elimination.

Reviewer: 2
Dr. Paleeratana Wongrith , Walailak University

Comments to the Author:	Authors' response
Overall assessment<br>Thanks to the editors for the opportunity to review this well-structured cross-sectional manuscript. This is a population study examining associations between healthy life expectancy (HLE) and area-level risk factors within one English Integrated Care System (ICS). The topic is policy-relevant and methodologically grounded in publicly available data sources. The manuscript adheres broadly to STROBE reporting standards and is clearly written.	Thank you

However, several methodological clarifications and analytical justifications are required to strengthen internal validity, interpretability, and reproducibility.	
1. BACKGROUND / INTRODUCTION<br>Strengths<br>• The scientific rationale is clearly stated (Introduction paras 1–2).<br>• The policy relevance of estimating HLE at the small-area level is well justified.<br>• The objective is explicitly defined (Introduction para 3).<br>• The study design is clearly indicated in the title and abstract as cross-sectional.<br>• The abstract is generally balanced and informative.<br>Issues/Points for Clarification<br>1. Conceptual framework<br>o The introduction would benefit from a clearer conceptual model explaining the assumed pathway between area-level risk factors and HLE.<br>o At present, risk factors appear selected based on availability and evidence, but their interrelationships (e.g., deprivation, income, and behavioral risk clustering) are not explicitly theorized.	Thank you.<br>We have now clarified that the risks are those linked to poor health outcomes. Lines 101-107 now read:<br>'*Previous analyses have found associations between shorter life expectancy and risk factors at national level, including higher alcohol intake, higher rates of smoking and obesity, and physical inactivity [8, 10-12], but there have been no recent analyses of such associations at small area level. There is therefore a need for local organisations to be able to estimate HLE for small areas more recently than 2013, and to compare local HLE with locally varying levels of exposure to health risks that are known to be linked to poor heath outcomes.'*<br>The selection of specific risk factors is covered in the methods section. We agree about the importance of the interrelationships between risk factors and have added that:<br>'*We also considered household income and percentage of older adults living alone to be complex indicators of social position that could affect multiple other health-related factors, acting at individual, household and societal levels.*' (lines 173-175)
2. Ecological inference<br>o The ecological nature of inference (MSOA-level data) should be stated explicitly earlier in the Introduction.<br>o A brief acknowledgment of ecological fallacy would strengthen the framing.	We have described the study as 'ecological' in a new sentence at the start of the introduction:<br>'*This cross-sectional ecological study uses a standard approach to estimating healthy life expectancies in MSOAs in Norfolk and Waveney.*' (lines 119-120)<br>We have added the following limitation:<br>'*All estimates refer to MSOA populations and cannot be assumed to apply to all individuals within those population groups.*' (lines 380-381)
3. Justification for ICS focus<br>o The rationale for focusing on a single ICS is implied but could be strengthened by clarifying why this ICS is particularly informative (e.g., diversity, deprivation gradient, rural–urban mix).	We have amended the relevant sentence to read:<br>'*This geographical area, with a range of deprivation and a mix of rural and urban areas, was is used as an example of how local information on HLE and risk factor exposure can be estimated and compared.*' (lines 112-114)

2. METHODS<br>Strengths<br>• The study design is clearly stated (cross-sectional population-based study).<br>• Setting and population are clearly described (128 MSOAs, 1,032,500 people).<br>• Ethical approval is appropriately documented.<br>• Data sources for risk factors are transparently described, including spatial methods for PM2.5.<br>• Funding statement and conflicts of interest are clearly reported.	Thank you
Major Methodological Comments<br>1. Study size and statistical power<br>The manuscript states the study size (128 MSOAs), but there is:<br>• No formal discussion of statistical power.<br>• No consideration of model overfitting relative to the number of predictors.<br>Given 10 candidate predictors and 128 area-level observations, please justify:<br>• Variable selection approach.<br>• Whether multicollinearity was assessed (e.g., VIF).<br>• How overfitting risk was mitigated.<br>Reference 24 suggests awareness of variable selection methodology, but the operational strategy must be clearly described in Methods.	Thankyou.<br>We have added this comment on sample size to the methods (lines 202-205):<br>'*We did not do prior sample size or power calculations because the sample size was pre-determined and there was no previous evidence to inform assumptions about the magnitude of parameters required for the calculations*.'<br>The sample size was sufficient to identify several statistically significant associations.<br><br>We have removed the stepwise elimination of variables.<br><br>We have added an assessment of multicollinearity using VIF and added this to the methods:<br>'To assess collinearity of risk factors we estimated variance inflation factors (VIFs) after each regression model' (lines 195-197).<br>These results are reported in Table S5, and summarised in the text of Results; we have added *'These VIFs indicate that collinearity between covariates may have contributed to some associations being non-significant.'* (lines 286-288)<br><br>We recognise that the regression models, with 128 observations and 9 covariates, could be at risk of overfitting, and that this could potentially be addressed by use of penalised, ridge or risk regression, and a range of regression diagnostic methods. However we consider this risk to be small, considering that the number of observations per covariate is 14 (128/9), which is

	more than the conventionally accepted level of 10. Give the range and complexity of the analyses and results already reported in this articile we considered it inappropriate to further extend this analysis.
2. Handling of missing data<br>STROBE item 12c indicates missing data handling is discussed in Discussion.<br>but:<br>• Missingness procedures should be described in Methods, not solely in Discussion.<br>• Please clarify:<br>o Were there missing MSOAs? NO<br>o Were complete-case analyses used? N/A<br>o Were any imputations performed? N/A	We have added this sentence to the methods:<br>'*Risk factors were excluded if accurate data were not available*.' (lines 184-185)<br><br>There were no missing MSOAs.<br><br>Complete-case analyses and imputations were not applicable here.
3. Risk factor temporal alignment<br>Several datasets are not from 2021 (e.g., income 2019–20).<br>This introduces:<br>• Potential temporal mismatch bias.<br>• Risk of misclassification if area conditions changed.<br>Please:<br>• Quantify time gaps for each exposure.<br>• Provide sensitivity analysis (if feasible).<br>• Justify why this does not materially affect conclusions.	Information on risk factors, data sources and dates is in the Supplementary material and Table S1.<br>We have added this information to the beginning of the methods section:<br>*'This cross-sectional study uses a standard approach to estimating healthy life expectancies in MSOAs in Norfolk and Waveney. Associations between HLEs and risk factors for poorer health and mortality were estimated using correlation coefficients and linear regression. Data pertain to 2017 to 2021.'* (lines119-122).<br><br>We have added a sensitivity analysis, described in the methods:<br>'*As a sensitivity analysis, because household income was most strongly associated with HLEs, and was also strongly associated with several risk factors, we repeated the four regression models, omitting income as a covariate*.' (lines 197-199)
4. Multicollinearity and deprivation<br>Index of Multiple Deprivation (IMD) and income are conceptually overlapping variables.<br>Please clarify:<br>• Were correlation matrices examined?<br>• Was IMD included alongside income in the same model?<br>• How was redundancy handled?<br>This is particularly important because deprivation indices may capture several	Thankyou. Yes, we have added correlations in Supplementary Table S4.<br>IMD was not included alongside income in the same model, in order to avoid collinearity.<br>We agree that deprivation 'captures' other risk factors, and have now included a sensitivity analysis, described as follows:<br>'*As a sensitivity analysis, because household income was most strongly associated with HLEs, and was also strongly associated with several risk factors, we repeated the four regression models, omitting income as a covariate'* (lines 197-199)

of the other included risk factors.	
5. Model specification clarity<br>The abstract reports regression coefficients (e.g., income coefficient 0.02).<br>but:<br>• The unit of change must be explicitly stated (e.g., per £1? per £100? per SD?).<br>• It is unclear whether predictors were standardized.<br>• Clarify whether coefficients are linear and whether assumptions (normality, homoscedasticity) were tested.	The units are stated in the table and text, including the abstract.<br>Predictors were not standardised and the regression table makes it clear that they werent.<br>We have added this to the methods:<br>'*We examined scatter graphs for heteroscedasticity, and examined histograms of HLEs for normality*.' (lines 185-186).<br>We have added this to the results:<br>'*Scatter graphs and histograms of HLEs showed approximate heteroscedasticity and normality respectively*.' (lines 274-275)
6. Ecological bias<br>As this is an ecological analysis at the MSOA level:<br>• Individual-level causal interpretation must be avoided.<br>• An explicit statement on ecological fallacy should be included in Methods and Discussion	Thankyou. See point 2 above:<br>We have described the study as 'ecological' in a new sentence at the start of the introduction:<br>'*This cross-sectional ecological study uses a standard approach to estimating healthy life expectancies in MSOAs in Norfolk and Waveney*.' (lines 119-120)<br>We have added the following limitation:<br>'*All estimates refer to MSOA populations and cannot be assumed to apply to all individuals within those population groups*.' (lines 380-382)
7. Sensitivity analyses<br>STROBE indicates no sensitivity analyses were performed.<br>Given:<br>• Risk factor selection decisions,<br>• Potential collinearity,<br>• Temporal mismatch,<br>At least one sensitivity model (e.g., removing IMD or testing alternative model forms) would strengthen robustness.	Thank you for this suggestion. See point 3 above:<br>We have added a sensitivity analysis, described in the methods:<br>'*As a sensitivity analysis, because household income was most strongly associated with HLEs, and was also strongly associated with several risk factors, we repeated the four regression models, omitting income as a covariate*.' (lines 197-199)<br>We have amended the STROBE document to include this.
3. RESULTS<br>Strengths<br>• Clear reporting of HLE ranges and area differences<br>• Regression coefficients reported with confidence intervals.<br>• Tables referenced appropriately, T1 about outcome variable in ecological outcome indicator.	Thank you.
Points Requiring Clarification<br>1. Interpretation of coefficient magnitude<br>The abstract reports an income coefficient 0.02 (95% CI 0.01–0.02).	We have now included this in the abstract:<br>'*Higher income was strongly associated with all healthy life expectancies: £100 higher weekly income was associated with 4.4 (95% confidence*

However:<br>• The practical meaning of 0.02 years per unit income is unclear.<br>• Consider translating into meaningful increments (e.g., per £1,000 weekly income increase).<br>• Consider presenting predicted HLE differences across deprivation quintiles.	*limits 3.5,5.2) and 4.6 (3.8,5.4) years greater HLE at birth in males and females respectively.'* (lines 46-48).<br>We have not presented predicted HLE differences across deprivation quintiles due to space limitations in this paper.
2. Sex-stratified analysis<br>Results focus primarily on males aged 65 in the abstract.<br>Please clarify:<br>• Were models run separately for men and women?<br>• Why are female regression results not equally presented?<br>• Were interaction terms tested?	Yes, models were run separately for men and women, and the female regression results are equally presented. We now present the female results separately in Table 3, and the male results in Table 4. Interaction terms were not tested.
3. Multiple comparisons<br>With 10 candidate risk factors:<br>• Was adjustment for multiple testing considered?<br>• Clarify selection pathway to final model.	Thankyou. We did not adjust for multiple testing as we do not think this would help interpretation of these results.<br><br>We have removed the stepwise elimination.
4. Spatial autocorrelation<br>Given MSOA-level data:<br>• Were spatial dependencies tested (e.g., Moran's I)?<br>• If spatial clustering exists, OLS assumptions may be violated.<br>Given spatial methods were used in exposure construction, spatial dependence should be considered analytically.	We have added this limitation:<br>'*Spatial dependencies were not tested*.' (lines 381-382)
4. DISCUSSION<br>Strengths<br>• Key findings summarized clearly.<br>• Strengths and limitations section included<br>• Funding independence stated	Thank you
Areas for Strengthening<br>1. Causal language<br>Some phrasing implies directional impact (e.g., prioritise interventions based on associations).<br>Given a cross-sectional ecological design:	Thank you<br><br>We now say:<br>'*...our results show varying associations between risk factors and outcomes in different small areas but cannot show causation*.' (lines 375-377),<br>and have added:

• Emphasize association rather than causation.<br>• Clarify that reverse causality cannot be excluded.	'*In some instances such as falls, income and poor health, reverse causality cannot be excluded.*' (lines 377-378).
2. Magnitude of potential bias<br>STROBE recommends discussing direction and magnitude of bias.<br>Expand discussion of:<br>• Measurement error in behavioral exposures (diet, physical inactivity).<br>• Ecological misclassification.<br>• Temporal mismatch bias.<br>• Unmeasured confounding (e.g., smoking).	Thank you. We say:<br>'*If variables were not measured accurately their estimated associations will tend to be biased towards showing no association. For this reason, we excluded some publicly available data*' (lines 384-385).<br>And:<br>'*Our data are cross-sectional and although risk factors can have short-term consequences, many have influences on health over decades that might not be fully captured with these data*' (lines 378-380).<br>We have added:<br>'*More accurate data on risk exposures in small areas is needed, particularly for smoking and also for other behavioural exposures such as diet and physical inactivity.*' (lines 394-396).<br>And:<br>'*Exclusion of smoking data may have led to some unmeasured confounding.*' (lines 393-394)
3. External validity<br>Generalizability is briefly addressed.<br>Please clarify:<br>• Is this ICS representative of England?<br>• Would results differ in urban metropolitan ICS areas?	This ICS is not statistically representative of England, but it has a range of deprivation and a mix of rural and urban areas that make it a useful example. The results are likely to differ in magnitude in urban metropolitan ICS areas.
4. Policy implications<br>The manuscript appropriately highlights local planning utility.<br>However:<br>• Include caution regarding overinterpretation.<br>• Consider discussing cost-effectiveness uncertainty of proposed interventions.	We have expanded the limitations as described above, to be cautious regarding overinterpretation.<br>We agree about the importance of considering cost-effectiveness, and have included evidence about cost-effectiveness of proposed interventions in paragraphs in the discussion (lines 422-448).
Conclusion<br>This is a strong, policy-relevant ecological study using publicly available data. The manuscript would be suitable for publication after moderate revision, primarily addressing:<br>1. Statistical model transparency and justification<br>2. Multicollinearity and spatial considerations	Thank you.<br>We have addressed these concerns as described above.

3. Missing data and sensitivity analyses<br>4. Clearer articulation of ecological limitations<br>Once strengthened analytically, this paper will provide valuable methodological guidance for local public health intelligence units.	

VERSION 2 - REVIEW

**Reviewer**	**1**
**Name**	**Dack, Kyle**
**Affiliation**	**University of Bristol, MRC Integrative Epidemiology Unit**
**Date**	**21-Jun-2026**
**COI**	

I appreciate the substantial revisions the authors have made, which strengthen the clarity of the methods/results, and address the concern I had regarding the modelling. I have no further suggestions or questions - the paper looks good.
